# Comparative genomic mapping reveals mechanisms of chromosome diversification in *Rhipidomys* species (Rodentia, Thomasomyini) and syntenic relationship between species of Sigmodontinae

**DOI:** 10.1371/journal.pone.0258474

**Published:** 2021-10-11

**Authors:** Vergiana dos Santos Paixão, Pablo Suárez, Willam Oliveira da Silva, Lena Geise, Malcolm Andrew Ferguson-Smith, Patricia Caroline Mary O’Brien, Ana Cristina Mendes-Oliveira, Rogério Vieira Rossi, Julio Cesar Pieczarka, Cleusa Yoshiko Nagamachi

**Affiliations:** 1 Laboratório de Citogenética, Centro de Estudos Avançados da Biodiversidade, Instituto de Ciências Biológicas, Universidade Federal do Pará (UFPA), Belém, Pará, Brazil; 2 Instituto de Biologia Subtropical (CONICET-UNAM), Puerto Iguazú, Misiones, Argentina; 3 Laboratório de Mastozoologia, Departamento de Zoologia, Universidade do Estado do Rio de Janeiro (UERJ), Rio de Janeiro, Brazil; 4 Cambridge Resource Centre for Comparative Genomics, Department of Veterinary Medicine, University of Cambridge, Cambridge, United Kingdom; 5 Laboratório de Zoologia e Ecologia de Vertebrados, Instituto de Ciências Biológicas, Universidade Federal do Pará (UFPA), Belém, Pará, Brazil; 6 Departamento de Biologia e Zoologia, Instituto de Biociências, Universidade Federal do Mato Grosso (UFMT), Cuiabá, Mato Grosso, Brazil; Universidade Federal de Ouro Preto, BRAZIL

## Abstract

*Rhipidomys* (Sigmodontinae, Thomasomyini) has 25 recognized species, with a wide distribution ranging from eastern Panama to northern Argentina. Cytogenetic data has been described for 13 species with 12 of them having 2n = 44 with a high level of autosomal fundamental number (FN) variation, ranging from 46 to 80, assigned to pericentric inversions. The species are grouped in groups with low FN (46–52) and high FN (72–80). In this work the karyotypes of *Rhipidomys emiliae* (2n = 44, FN = 50) and *Rhipidomys mastacalis* (2n = 44, FN = 74), were studied by classical cytogenetics and by fluorescence *in situ* hybridization using telomeric and whole chromosome probes (chromosome painting) of *Hylaeamys megacephalus* (HME). Chromosome painting revealed homology between 36 segments of REM and 37 of RMA. We tested the hypothesis that pericentric inversions are the predominant chromosomal rearrangements responsible for karyotypic divergence between these species, as proposed in literature. Our results show that the genomic diversification between the karyotypes of the two species resulted from translocations, centromeric repositioning and pericentric inversions. The chromosomal evolution in *Rhipidomys* was associated with karyotypical orthoselection. The HME probes revealed that seven syntenic probably ancestral blocks for Sigmodontinae are present in *Rhipidomys*. An additional syntenic block described here is suggested as part of the subfamily ancestral karyotype. We also define five synapomorphies that can be used as chromosomal signatures for *Rhipidomys*.

## Introduction

The Sigmodontinae subfamily is the most diverse and complex group of cricetid rodents in the New World, with 74 genera and 380 species grouped in 10 tribes (Abrotrichini, Akodontini, Ichthyomyini, Oryzomyini, Phyllotini, Reithrodontini, Sigmodontini, Thomasomyini, Wiedomyini, Euneomyini), and 12 genera not included in any of the mentioned tribes (*incertae sedis*) [1–4].

**Fig 1:**
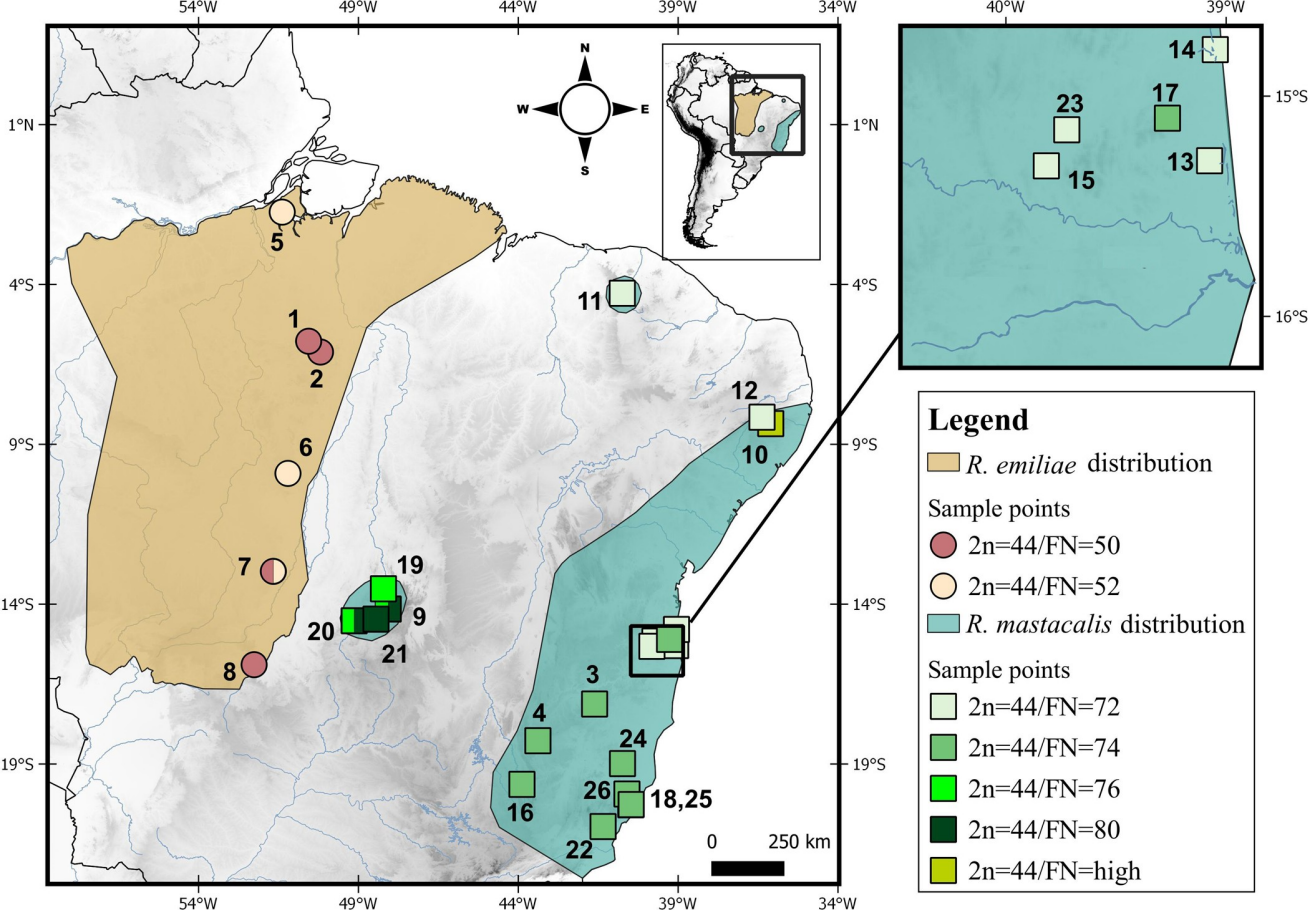
Map showing the distribution areas and collection points of *R*. *emiliae* and *R*. *mastacalis* with cytogenetic data available in the literature, and the specimens karyotyped in the present study (localities 1–4). The distribution data were extracted from the literature [6, 7]. Other information from the literature is shown in Table 1. The diploid number (2n) and autosomal fundamental number (FN) are based on the information in Table 1. The numbers refer to localities in Tables 1 and 2. The database was obtained from DIVA-GIS [13].

The genus *Rhipidomys* Tschudi, 1845 is the only arboreal representative of the Thomasomyini tribe [1, 5, 6]. Twenty-five species are currently recognized, with a high degree of morphological similarity that comprises a taxonomically complex group [1, 5–9]. The species of this genus range in length from 90 mm to 210 mm, are difficult to capture due to their arboreal habits, and are among the least well-known species in the Neotropical region [5, 6, 10]. These rodents occur from eastern Panama and along South America up to northern Argentina [6]. However, the geographical limits of *Rhipidomys* species are poorly understood [11]. Eleven species occur in the Brazilian biomes [6], among them, *Rhipidomys emiliae* Allen 1916 is distributed in the Amazon biome and the Amazon-Cerrado ecotone, from Pará to Mato Grosso; *Rhipidomys mastacalis* Lund 1840 is found in the Atlantic Forest biome and also in central Brazil [6, 11] (Fig 1). These two species form a clade together with the Cerrado species, *R*. *ipukensis* Rocha, Costa & Costa, 2011. This latter species is closely related to *R*. *emiliae*; together, they form a sister group with *R*. *mastacalis* [7, 12].

Studies using classical cytogenetics in *Rhipidomys* reveal that most of the analyzed species have the same diploid number (2n = 44). The autosomal fundamental number (FN), varies from 46 to 80; the exception known to date is *R*. *nitela* with 2n = 48/FN = 67/68 and 2n = 50/FN = 71/72 (Tables 1 and S1). In the species with 2n = 44, two karyotypic patterns are observed: karyotypes with low FN (46, 48, 49, 50, 52), with *R*. *emiliae* being one of the representatives of this group; and karyotypes with high FN (72, 74, 76, 80), represented only by *R*. *mastacalis* (Table 1).

**Table 1:** Cytogenetic data available in the literature and obtained in the present study for *R*. *emiliae* and *R*. *mastacalis*.

Species	2n	FN	Locality	References
*R*. *emiliae*	44	50	(1) BR: PA, Marabá, FLONA de Tapirapé-Aquiri	**Present work**
*R*. *emiliae*	44	50	(2) BR: PA, Parauapebas, FLONA de Carajás	**Present work**
*R*. *emiliae* ^1^	44	52	(5) BR: PA, Melgaço, FLONA de Caxiuanã	[14]
*R*. *emiliae* ^2^	44	52	(6) BR: MT, Vila Rica	[15]
*R*. *emiliae*	44	52	(7) BR: MT, Ribeirão Cascalheira	[16]
*R*. *emiliae* ^3^	44	50	(7) BR: MT, Ribeirão Cascalheiras, Fazenda Noruimbá	[17]
*R*. *emiliae*	44	52	(8) BR: MT, Barra do Garças	[16]
*R*. *mastacalis*	44	74	(3) BR: MG, Fazenda Palmares, Padre Paraíso	**Present work**; [18]
*R*. *mastacalis*	44	74	(4) BR: MG, Diamantina, Fazenda Santa Cruz,	**Present work**; [18]
*R*. *mastacalis* cytotype 2	44	76	(9) BR: GO, Serra da Mesa, 20 km NW Colinas do Sul	[14]
*R*. *mastacalis* cytotype 1	44	80	(9) BR: GO, Serra da Mesa, 20 km NW Colinas do Sul	[14]
*R*. *mastacalis* ^4^	44	high	(10) BR: PE, Serra dos Cavalos, 13 km ESE São Caitano	[19]
*R*. *mastacalis* ^5^	44	72	(11) BR: CE, Ipú, Serra de Ibiapaba	[20]
*R*. *mastacalis* ^5^	44	72	(12) BR: PE, Brejo da Madre de Deus, RPPN Fazenda Bituri	[20]
*R*. *mastacalis* ^5^	44	72	(13) BR: BA, Una, ReBio de Una	[20]
*R*. aff. *mastacalis* 1	44	72	(13) BR: BA, Una	[16]
*R*. *mastacalis* ^5^	44	72	(14) BR: BA, Ilhéus, Centro Experimental Almada	[20]
*R*. *mastacalis* ^5^	44	72	(15) BR: BA, Jussari, RPPN Serra do Teimoso	[20]
*R*. *mastacalis*	44	74	(16) BR: MG, Lagoa Santa	[19]
*R*. *mastacalis*	44	74	(17) BR: BA, Una, Fazenda Unacau, 8km São José	[19]
*R*. *mastacalis*	44	74	(18) BR: ES, Cariacica, ReBio Duas Bocas	[21]
*R*. *mastacalis* cytotype 2	44	76	(19) BR: GO, Serra da Mesa, 40 km SW Minaçú	[14]
*R*. *mastacalis* cytotype 2	44	76	(20) BR: GO, Serra da Mesa, 40 km NE Uruaçú	[14]
*R*. *mastacalis* cytotype 1	44	80	(20) BR: GO, Serra da Mesa, 40 km NE Uruaçú	[14]
*R*. *mastacalis* cytotype 2	44	76	(21) BR: GO, Serra da Mesa, 55 km N Niquelândia	[14]
*R*. *mastacalis*	44	74	(22) BR: ES, Muqui, Fazenda Recanto	[16, 17]
*R*. aff. *mastacalis* 1	44	72	(23) BR: BA, Itajú do Colônia	[16]
*R*. *mastacalis*	44	74	(24) BR: ES, Águia Branca	[16, 17]
*R*. *mastacalis*	44	74	(25) BR: ES, Cariacica, ReBio Duas Bocas	[16, 17]
*R*. aff. *mastacalis* 2	44	74	(26) BR: ES, Santa Tereza	[16]

The numbers in parentheses refer to localities mentioned in Fig 1. Abbreviations: Brazil (BR). Brazilian States: Bahia (BA), Ceará (CE), Goiás (GO), Espírito Santo (ES), Mato Grosso (MT), Minas Gerais (MG), Pará (PA), Pernambuco (PE), and Piauí (PI); diploid number (2n); autosomal fundamental number (FN); National Forest (FLONA); Natural Heritage Private Reserve (RPPN); and Biological Reserve (ReBio).

^**1**^ Identified in the original article as *R*. *leucodactylus* cytotype 1 and reviewed by Tribe [6].

^**2**^ Identified in the original article as *Rhipidomys* cf. *mastacalis* and reviewed by Tribe [6].

^**3**^ Identified in the original study as *Rhipidomys* sp., however Costa et al. [7] and Tribe [6] assign the collecting locality of the specimen to the range of *R*. *emiliae*.

^**4**^ Identified in the original article as *R*. *cearanus*, however this name is currently considered a synonym of *R*. *mastacalis* [1].

^**5**^ Previously assigned as 2n = 44/FN = 70 [20], but we corrected this to 2n = 44/FN = 72 [16]. The chromosome morphology and size in relation to the other karyotype pairs and the G-Banding pattern allowed us to conclude that the chromosome identified as autosome pair 16 (acrocentric) was actually the sex X chromosome, and the chromosome that was defined as X (submetacentric) has the G-banding pattern and chromosomal morphology similar to the autosome RMA 8.

In this genus, pericentric inversions have been identified as the main cause of the variation in the number of acrocentric versus bi-armed chromosomes, especially when species with high FN are compared with those with low FN [11, 12, 16, 19, 20]. In karyotypes with 2n other than 44 (from the *R*. *nitela* group [14]), fusions/fissions or translocations rearrangements have been suggested [14, 15, 22].

The rodent genome shows great variability in diploid numbers and chromosomal morphology, both between and within species [23]. In this Order, diploid numbers range from 10 in *Ctenomys steinbachi* (Ctenomyidae) [24] and *Akodon* sp. (Sigmodontinae) [25, 26] to 118 in *Dactylomys boliviensis* (Echimyidae) [27]. This variability in chromosome numbers can result from Robertsonian translocations (centric fusion and fission), *in tandem* fusions, or from a variable number of B chromosomes. The variation of chromosomal morphology can also result from pericentric inversions, reciprocal translocations or centromeric repositioning, or from variation in constitutive heterochromatin [23]. Considering the chromosomal diversity observed in rodents, any hypothesis about the origin and evolution of their chromosomes depends on the analysis of conserved syntenies between species. Classical cytogenetics combined with chromosome painting is a useful approach in comparing these karyotypes [28–32]. As a consequence, knowledge about the karyotypic evolution of some groups of rodents has been expanded significantly, such as in species representing the three tribes of Sigmodontinae: using *Sigmodon* probes in eight species of *Sigmodon* (tribe Sigmodontini) [33]; using *Akodon* probes in four species of *Akodon* (tribe Akodontini) [34]; using *Mus musculus* probes (Family Muridae) in four species of *Akodon*, one species of *Necromys* and *Thaptomys* (tribe Akodontini) and one species of *Oligoryzomys* (tribe Oryzomyini) [35, 36]; using *Oligoryzomys* probes in seven species of *Oligoryzomys* (tribe Oryzomyini) [37]. In addition to these, whole chromosome probes from *Hylaeamys megacephalus* (HME, Oryzomyini tribe) [30] were developed for studies on comparative genomics in Sigmodontinae, and employed subsequently in six Akodontinini species belong to the genera *Akodon*, *Thaptomys*, *Necromys*, *Oxymycterus*, *Blarinomys* and in 13 Oryzomyini species referring to genera *Neacomys*, *Oecomys*, *Cerradomys* [30–32, 38–42]. Studying the karyotypes of representatives of the Akodontini and Oryzomyini tribes with HME probes allowed researchers to propose characters for the putative ancestral karyotype of the subfamily [32]. This is particularly relevant since HME (Oryzomyini) is phylogenetically close to Thomasomyini and Akodontini (S1 Fig). However, we currently lack chromosomal painting data for *Rhipidomys* or any other genus of the Thomasomyini tribe.

In the present study, we used chromosomal painting with HME whole chromosome probes [30] and G-banding to investigate chromosomal homologies between the karyotypes of *R*. *emiliae* (2n = 44/FN = 50) and *R*. *mastacalis* (2n = 44/FN = 74), and to determine if pericentric inversions are the predominant chromosomal rearrangements responsible for the karyotypic divergence between these species, which were selected as representative of the groups with low and high FN, respectively. We selected HME probes because they have already been used in several taxa of the Sigmodontinae [30–32, 38–42] and are phylogenetically close to the species studied here. This allowed us to compare the karyotype of the *Rhipidomys* with those previously hybridized with these probes, so that shared chromosomal characters could be identified.

## Material and methods

### Samples

We examined six specimens of *R*. *emiliae* trapped in two localities of the Pará state, Brazil, and four specimens of *R*. *mastacalis* from two municipalities in Minas Gerais state, Brazil (Fig 1).

The *Rhipidomys mastacalis* specimens were identified according to their morphological features and their external and craniodental measurements, and compared with specimens deposited in the mammal collection of the Museu Nacional (UFRJ, Rio de Janeiro). *Rhipidomys mastacalis* can be identified by the pelage coloration–gray-brown to more red-brown in the dorsal portion [6]. Cranial characteristics, such as rostrum length, straight supraorbital ridges, not greatly inflated or rounded braincase were also considered for their identifying features as was the derived carotid circulatory pattern. Similarly, the *Rhipidomys emiliae* specimen was identified by the morphological features and by external and craniodental measurements [6]. This species has a dull grayish-brown to brighter orange-brown agouti dorsal pelage and cream or white ventral pelage, and has craniodental characters such as moderately developed supraorbital ridges diverging posteriorly from a point well forward, resulting in a broad interorbital region, upper toothrow length varying from 4.5 to 5.1 mm, incisive elliptical shaped foramina, and a derived carotid circulatory pattern.

Information about the samples is summarized in Table 2. The skulls and skins used to identify the species are deposited in the mammal collection of the Museu Paraense Emilio Goeldi (MPEG), Museu de Zoologia da Universidade Federal do Pará (MZUFPA), and Museu Nacional, Universidade Federal do Rio de Janeiro (MN).

**Table 2:** Samples and collection localities of *R*. *emiliae* and *R*. *mastacalis*.

Species	2n/FN	Locality	Geographic coordinate	Museum Number
*R*. *emiliae*	44/50	(1) BR: PA, Tapirapé-Aquiri National Forest, Marabá	05°46’21”S, 50°33’21”W	MPEG 40563 ♂
		(2) BR: PA: Carajás National Forest, Parauapebas	06°05’49”S, 50°08’34”W	MZUFPA CAR 41 ♀
			06°05’49”S, 50°08’34”W	MZUFPA CAR 60 ♂
			06°05’49”S, 50°08’34”W	MZUFPA CAR 275 ♂
			06°05’40”S, 50°07’15”W	MZUFPA CAR 231 ♂
			06°05’52”S, 50°07’55”W	MZUFPA CAR 268 ♂
*R*. *mastacalis*	44/74	(3) BR: MG: Fazenda Palmares, Padre Paraíso	17°7’16.6”S, 41°36’37.6”W	MN 82897 ♀
				MN 82899 ♂
				MN 82900 ♀
		(4) BR: MG, Fazenda Santa Cruz, Diamantina	18°16’11.6”S, 43°23’4.2”W	MN 82896 ♂

Abbreviations: Diploid number (2n); fundamental number (FN); Brazil (BR); state of Pará (PA); state of Minas Gerais (MG). Symbols: male (♂) and female (♀). The numbers in parentheses refer to the localities mentioned in Fig 1.

The acronym CAR refers to the field number of specimens collected in the Carajás National Forest that will be deposited in the MZUFPA.

The specimens were collected following procedures recommended by the American Mammal Society. JCP and LG have permanent field licenses (numbers 13248 and 598633) from the “Chico Mendes Institute for Biodiversity Conservation”. The CEABIO Cytogenetics Laboratory at UFPA has authorization from the Ministry of the Environment for the transportation of samples (number 19/2003) and the use of samples for research (number 52/2003). This research was approved by the Ethics Committee of the Federal University of Pará (Permission 68/2015). Animals were euthanized using intraperitoneal injection of barbiturates (pentobarbital, 120 mg/kg) after local anesthesia (lidocaine used topically).

### Cytogenetic analysis

Chromosomal preparations were obtained from bone marrow [43] and G-banding [44], C-banding [45] fluorescent *in situ* Hybridization (FISH) with human telomeric probes (All Telomere, ONCOR) and chromosome painting [30] were performed according to the described protocols. At least ten metaphases were analyzed in each sample by these techniques.

Chromosome painting with whole chromosome probes of *H*. *megacephalus* (HME; female; 2n = 54/FN = 62) [30] was performed as previously described [30, 46]. Of the 24 probes, 21 corresponded to individual chromosome pairs and three corresponded to two pairs (HME (9,10), HME (13,22), and HME (16,17)). Briefly, mitotic chromosomes preparations were denatured in 70% formamide/2× SSC at 65°C for 50 seconds. The HME probes were denatured for 15 minutes at 70°C. *In situ* hybridization was performed for 72 h at 37°C. After hybridization and washing the slides (2× formamide 50%, 2× 2SSC, 1× 4SSC/Tween at 40°C); biotinylated probes were detected with avidin-Cy3 (red) or avidin-FITC (green). The slides were counterstained with DAPI (4’,6-diamidino-2-phenylindole; blue) and we used inverted DAPI staining (G-banding pattern) for the correct assignment of the hybridized chromosomes. The digital images were captured using AxioVision 3.0 software and a CCD camera (AxioCam) coupled to a Zeiss-Axiophot 2 microscope or with the Nis-Elements software to a Nikon H550S microscope. Adobe Photoshop CS4 software was used for final image editing.

## Results

### Classical cytogenetics

**Fig 2:**
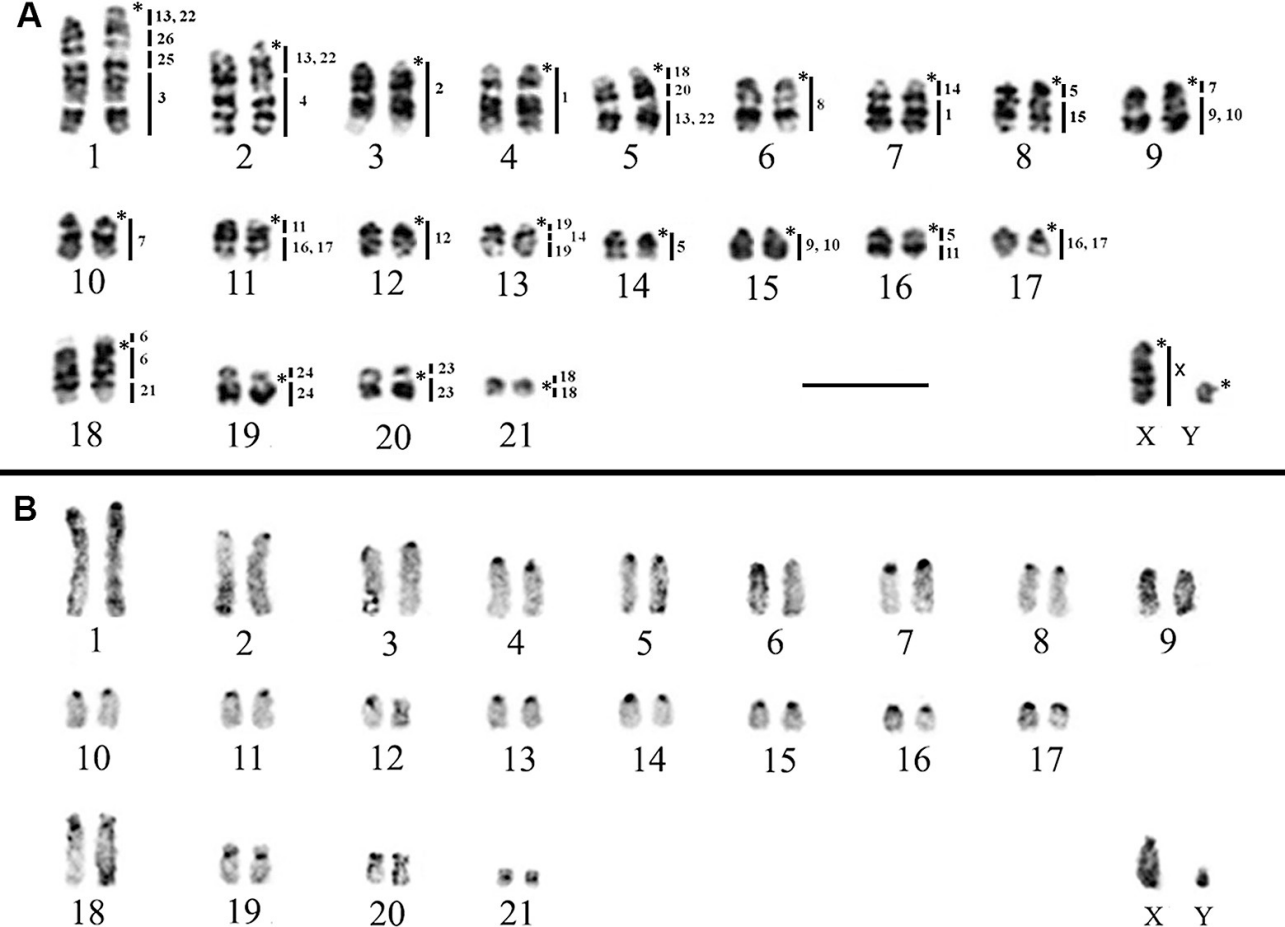
Karyotype of *R*. *emiliae* (2n = 44, FN = 50). A) G-banding with genomic mapping using whole chromosome probes of *H*. *megacephalus*. B) C-banding showing the locations of constitutive heterochromatin. (*) Indicates centromere. Bar: 10 μm.

*Rhipidomys emiliae* presents 2n = 44/FN = 50, with 17 autosomal acrocentric pairs (1–17) and four bi-armed pairs (18–21); the X chromosome is a medium acrocentric and the Y is a small acrocentric (Fig 2A). Constitutive heterochromatin (CH) was found to be equally distributed in the pericentromeric regions of all autosomal pairs and the X chromosome, whereas the Y chromosome is almost entirely heterochromatic, except for the short arm (Fig 2B).

**Fig 3:**
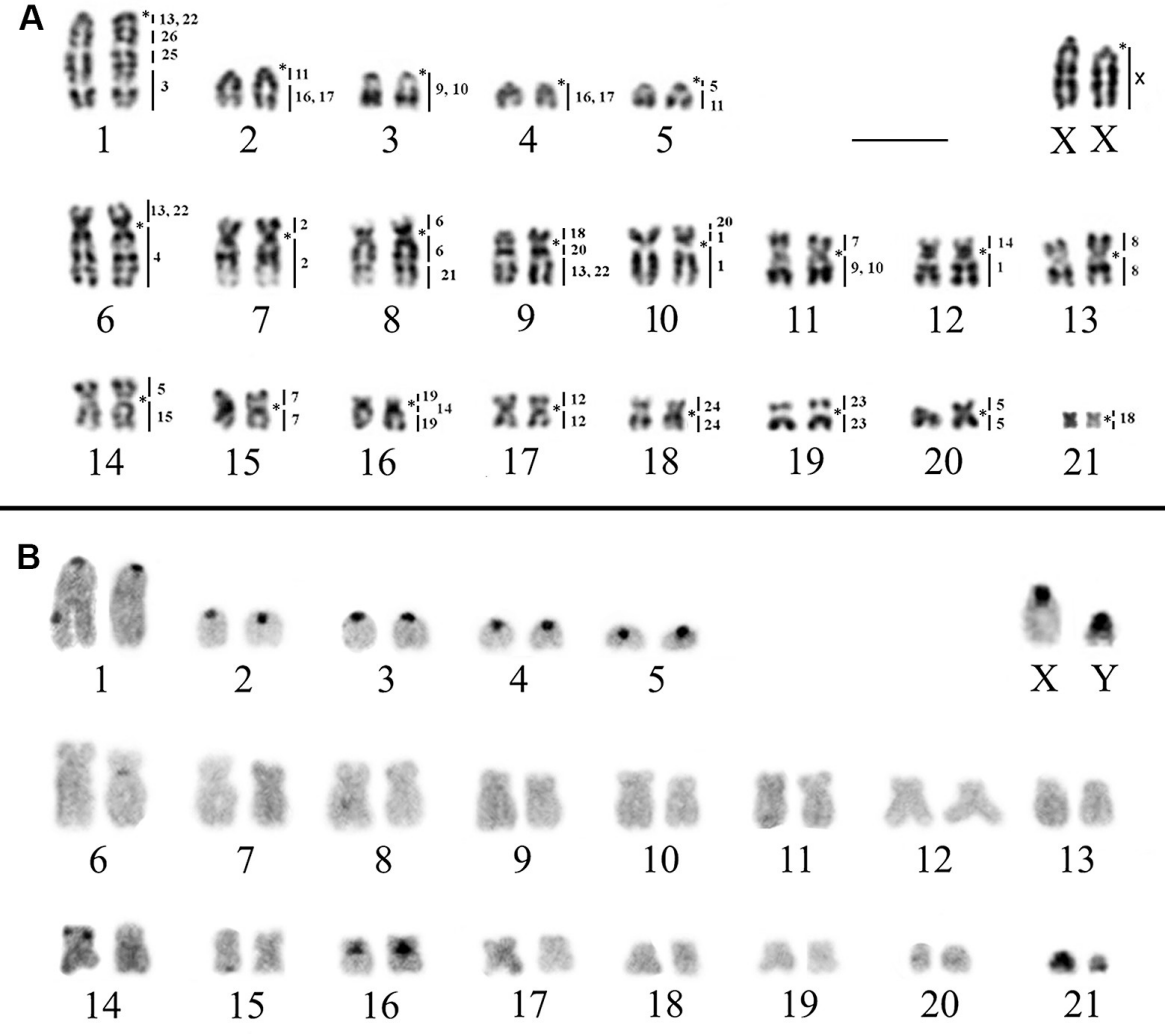
Karyotype of *R*. *mastacalis* (RMA, 2n = 44, FN = 74). A) G-banding with genomic mapping using whole chromosome probes of *H*. *megacephalus*. B) C-banding showing the location of the constitutive heterochromatin. (*) Indicates centromere. Bar: 10 μm.

The *R*. *mastacalis* karyotype (2n = 44/FN = 74) was previously published, but the information was restricted to the 2n and NF [18]. Here, we subjected this karyotype to chromosomal painting with HME probes in addition to chromosome banding. The samples revealed five autosomal acrocentric pairs (1–5) and 16 biarmed pairs (6–21); the X and Y chromosomes are medium and small acrocentrics, respectively (Fig 3A). The distribution of CH is not homogeneous: this is evident in the pericentromeric regions of the five acrocentric pairs and pair 16 (submetacentric); in pair 14 (submetacentric) it occurs in the distal region of the short arm of one of the homologs; in the other autosomal pairs it is less evident (in the centromeric region of pair 6) or not visible (in the other pairs); in the X chromosome, there is CH in the centromeric region; and the Y chromosome is almost entirely heterochromatic, except for the distal region of the long arm (Fig 3B).

### Fluorescence *in situ* hybridization (FISH)

**Fig 4:**
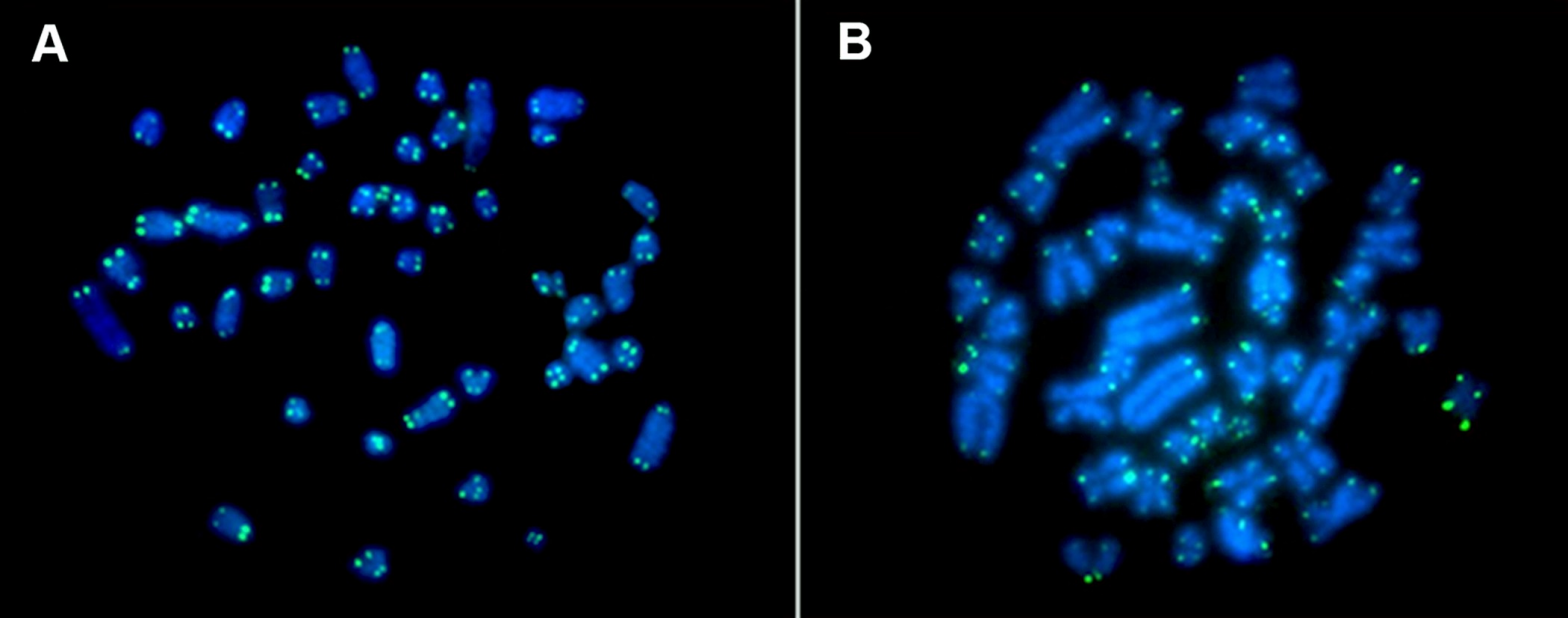
FISH with telomeric probes. (A) *R*. *emiliae*, (B) *R*. *mastacalis*.

FISH with telomeric probes showed only distal markings, and there was no evidence of ITSs (interstitial telomeric sequences) in the karyotypes of *R*. *emiliae* or *R*. *mastacalis* (Fig 4).

The hybridization analysis of the 24 HME probes in the karyotypes of *R*. *emiliae* (Fig 2A and Table 3) and *R*. *mastacalis* (Fig 3A and Table 3) revealed 36 and 37 regions of chromosomal homology, respectively.

**Table 3:** Number and localization of FISH signals observed in *R*. *emiliae* (REM) and *R*. *mastacalis* (RMA) karyotypes hybridized with whole chromosome probes of *Hylaeamys megacephalus* [30].

HME (2n = 54/FN = 62)	REM (2n = 44/FN = 50)		RMA (2n = 44/FN = 74)	
	N° of Signals	Chromosome localization	N° of Signals	Chromosomal localization
1	2	4, 7q distal	2	10p proximal+10q, 12q
2	1	3	1	7
3	1	1q distal	1	1q distal
4	1	2 q distal	1	6q
5	3	8q proximal, 14,16q proximal	3	5q proximal, 14p, 20
6	1	18p+q proximal	1	8p+8q proximal
7	2	9q proximal; 10	2	11p, 15
8	1	6	1	13
9,10	2	9q distal, 15	2	11q, 3
11	2	11q proximal; 16q distal	2	2q proximal, 5q distal
12	1	12	1	17
13,22	3	1q proximal, 2q proximal, 5q distal	3	1q proximal, 6p, 9q distal
14	2	7q proximal; 13q interstitial	2	12p, 16q proximal
15	1	8q distal	1	14q
16,17	2	11q distal, 17	2	2q distal, 4
18	2	5q proximal, 21	2	9p, 21
19	2	13q proximal, 13q distal (two different segments)	2	16p, 16q distal (two different segments)
20	1	5q interstitial	2	9q proximal, 10p distal
21	1	18q distal	1	8q distal
23	1	20	1	19
24	1	19	1	18
25	1	1q interstitial	1	1q interstitial
26	1	1q interstitial / proximal	1	1q interstitial
X	1	X	1	X
Total	36		37	

p = short arm; q = long arm.

### *Rhipidomys emiliae* (REM, 2n = 44/FN = 50)

In *R*. *emiliae*, 14 HME probes showed conserved synteny without any breaks. Among them, six probes (HME 2, 8, 12, 23, 24, and X) each corresponded to a pair of whole chromosomes (REM 3, 6, 12, 20, 19, and X, respectively), while eight (HME 3, 4, 6, 15, 20, 21, 25, and 26) hybridized to parts of chromosomes (REM 1q distal, 2q distal, 18p + q proximal, 8q distal, 5q interstitial, 18q distal, 1q interstitial, and 1q interstitial, respectively), in synteny with chromosomal regions that are homeologous to other HME probes (Table 3 and Fig 2A).

The remaining ten probes each exhibited more than one hybridization signal. Eight of them (HME 1, 7, (9,10), 11, 14, (16,17), 18, and 19) showed two signals each in *R*. *emiliae*, while probes HME 5 and HME (13,22) each showed three hybridization signals (Table 3 and Fig 2A).

**Fig 5:**
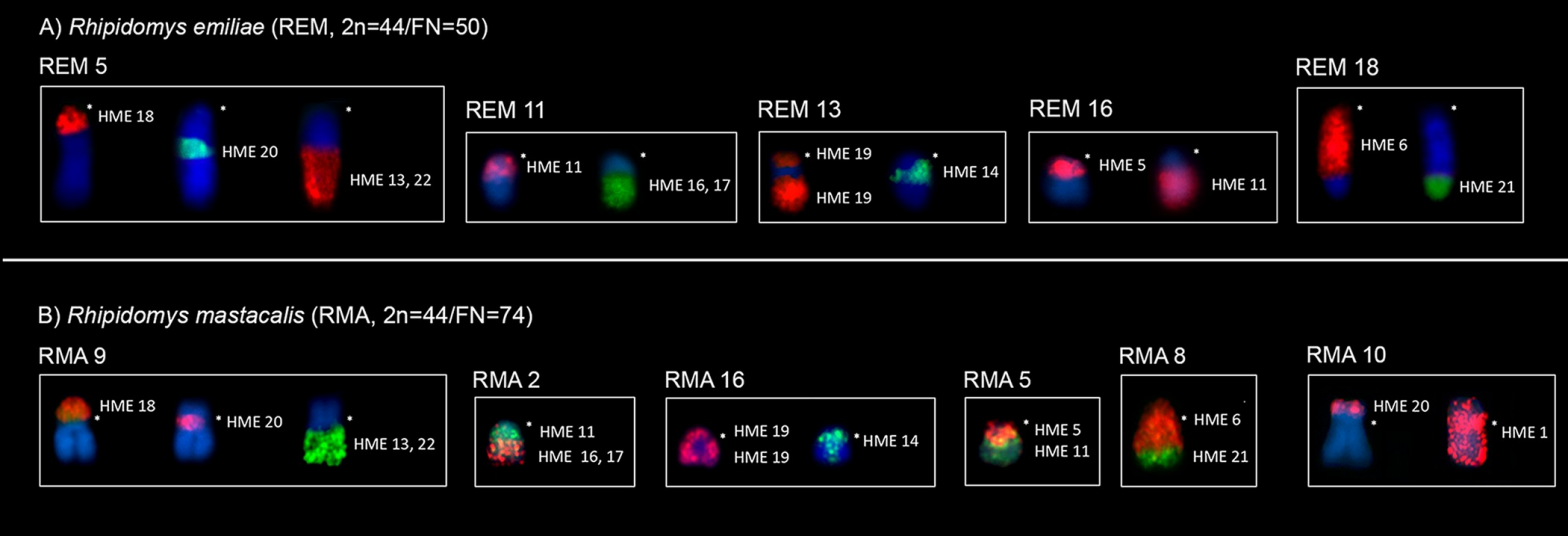
Chromosomal associations shared between (A) *R*. *emiliae* and (B) *R*. *mastacalis* using HME probes **(**REM 5 and RMA 9 / REM 11 and RMA 2 / REM 13 and RMA 16 / REM 16 and RMA 5 / REM 18 and RMA8). Additionally, the association HME1/HME20 (RMA 10) not found in REM is evident. Each box shows a chromosome pair with syntenic association. (*) Indicates a centromere.

Ten chromosomal pairs of *R*. *emiliae* showed hybridization signals with multiple HME probes (chromosomal associations): REM 1 (HME */(13,22)/26/25/3); REM 2 (HME */(13,22)/4); REM 5 (HME */18/20/(13,22); REM 7 (HME */14/1); REM 8 (HME */5/15); REM 9 (HME */7/(9,10)); REM 11 (HME */11/(16,17)); REM 13 (HME*/19/14/19); REM 16 (HME */ 5/11), and REM 18 (HME 6/*/6/21) (Figs 2A and 5A and Table 3).

### *Rhipidomys mastacalis* (RMA, 2n = 44/FN = 74)

In *R*. *mastacalis*, 13 HME probes produced unique hybridization signals and remained conserved, with no break in synteny. Of them, six (HME 2, 8, 12, 23, 24, and X) each hybridized to a single pair of whole chromosomes (RMA 7, 13, 17, 19, 18, and X, respectively), while seven (HME 3, 4, 6, 15, 21, 25, and 26) hybridized to parts of chromosomes (RMA 1q distal, 6q, 8p + 8q proximal, 14q, 8q distal, 1q interstitial, and 1q interstitial, respectively) associated in synteny with chromosomal regions that are homeologous to other HME probes (Table 3 and Fig 3A).

The remaining probes each showed more than one hybridization signal. Nine of them (HME 1, 7, (9,10), 11, 14, (16,17), 18, 19, and 20) yielded two signals each in *R*. *mastacalis*. Probes HME 5 and HME (13,22) both showed signals on three different chromosomes (Table 3 and Fig 3A).

Eleven chromosomal pairs of *R*. *mastacalis* showed chromosomal associations: RMA 1 (HME */(13,22)/26/25/3); RMA 2 (HME */11/(16,17)); RMA 5 (HME */5/11), RMA 6 (HME (13,22)/*/4); RMA 8 (HME 6/*/6/21); RMA 9 (HME 18/*/20/(13,22)); RMA 10 (HME 20/1/*/1); RMA 11 (HME 7/*/(9,10)); REM 12 (HME 14/*/1); RMA 14 (HME 5/*/15); and RMA 16 (HME 19/*/14/19) (Figs 3A and 5B and Table 3).

The S2 Fig shows metaphases with hybridizations of all the whole chromosomes probes of HME.

## Discussion

### Classical cytogenetic analysis

The karyotype formula found in this work for *R*. *emiliae* (2n = 44/FN = 50) is similar to that already described [17] (Fig 1, locality 7) but differs from the 2n = 44/FN = 52 [16] (Table 1 and Fig 1, localities 7 and 8) and for samples from Caxiuanã (Fig 1, locality 5) and Vila Rica (Fig 1, locality 6) [14, 15] reviewed [6] and considered as *R*. *emiliae*. This divergence between FN = 50 and 52 is due either to a pericentric inversion or to centromeric repositioning.

Karyotypes with 2n = 44/FN = 50 have been described for other species of *Rhipidomys* (*R*. *gardneri*, *R*. *itoan*, *Rhipidomys* cf. *macconnelli*, *R*. *macrurus*, *R*. *tribei*, and *Rhipidomys* sp.) (See S1 Table). Considering that many chromosomes of the genus are similar in size, it is not possible to define with certainty whether chromosomes with the same morphology are or are not homoeologous in the karyotypes of the different species. Thus, we are unable to determine whether the agreement of FN in different species indicates that they have similar karyotypes or are cases of homoplasy. Studies with chromosomal painting in these species may contribute more precisely to defining the homologies of the chromosomes involved in changing the FN.

In the present study, *R*. *mastacalis* was found to have a karyotype of 2n = 44/FN = 74. This is similar to some descriptions in the literature for this species [16–19, 21], while other authors [14, 16, 17, 20] have reported different values for FN (FN = 72, 76, and 80) (Table 1 and Fig 1). Considering the size of the chromosomes, we suggest that the difference between these karyotypes is related to chromosomes RMA 3–5 and RMA 21, which may have acrocentric or bi-armed morphology.

In the Atlantic forest, *R*. *mastacalis* presents two different karyotypes: to the south we find populations with FN = 74 (Table 1 and Fig 1, localities 3, 4, 16, 18, 22, 24–26), while northern populations have FN = 72 (Table 1 and Fig 1, localities 12–15 and 23). The populations found in isolated mesic forests (mesic enclaves or *brejos de altitude*) within the Caatinga domain of the northeastern region also have FN = 72 ([20] Fig 1, locality 11). If we assume a distinct evolution or biogeographic history for the *brejos de altitude* from northeastern Brazil [7], individuals isolated in these microenvironments provisionally identified as *R*. *mastacalis* probably represent specific entities; therefore, populations from this region should be further evaluated [6, 7]. The individuals isolated in the *brejos* of Serra da Ibiapaba (Table 1 and Fig 1, locality 11), which were provisionally identified as *R*. *mastacalis* due to FN = 70 (which we corrected for FN = 72, see Table 1) [20], may be elevated to species status (for which the named *R*. *cearanus* is available [6]) because of this distant basal position of a clade allying *R*. *emiliae* and *R*. *ipukensis* to which *R*. *mastacalis* forms the sister clade [17]. Other populations also need to be studied in detail, such as *Rhipidomys* specimens from northern Goiás state provisionally referred to *R*. *mastacallis* because of their high FN (FN = 76, 80) (Table 1 and Fig 1, localities 9, 12–21) [14] without reference to the morphological data of the specimens, which may correspond to *R*. *ipukensis* [6]. If this is confirmed, it would indicate that the high FN occurs not only in *R*. *mastacalis*, but also in *R*. *ipukensis* and *R*. *cearanus* (specimens from Serra da Ibiapaba) [6, 7]. The results of our C-banding analysis (Figs 2B and 3B) showed that there are differences in the amount and distribution of constitutive heterochromatin between *R*. *emiliae* and *R*. *mastacalis*. In the karyotype of specimens of *R*. *mastacalis* described [19], only the acrocentric pairs presented centromeric constitutive heterochromatin. In our samples, there was positive staining on some bi-armed chromosomes in addition to the acrocentric ones, indicating that there is intraspecific variation. These data demonstrate that the polymorphism of *Rhipidomys* cytotypes extends to heterochromatin and confirm that its addition/deletion is a common process in the genus [15, 47].

### Comparative mapping between *R*. *emiliae* and *R*. *mastacalis*

**Fig 6:**
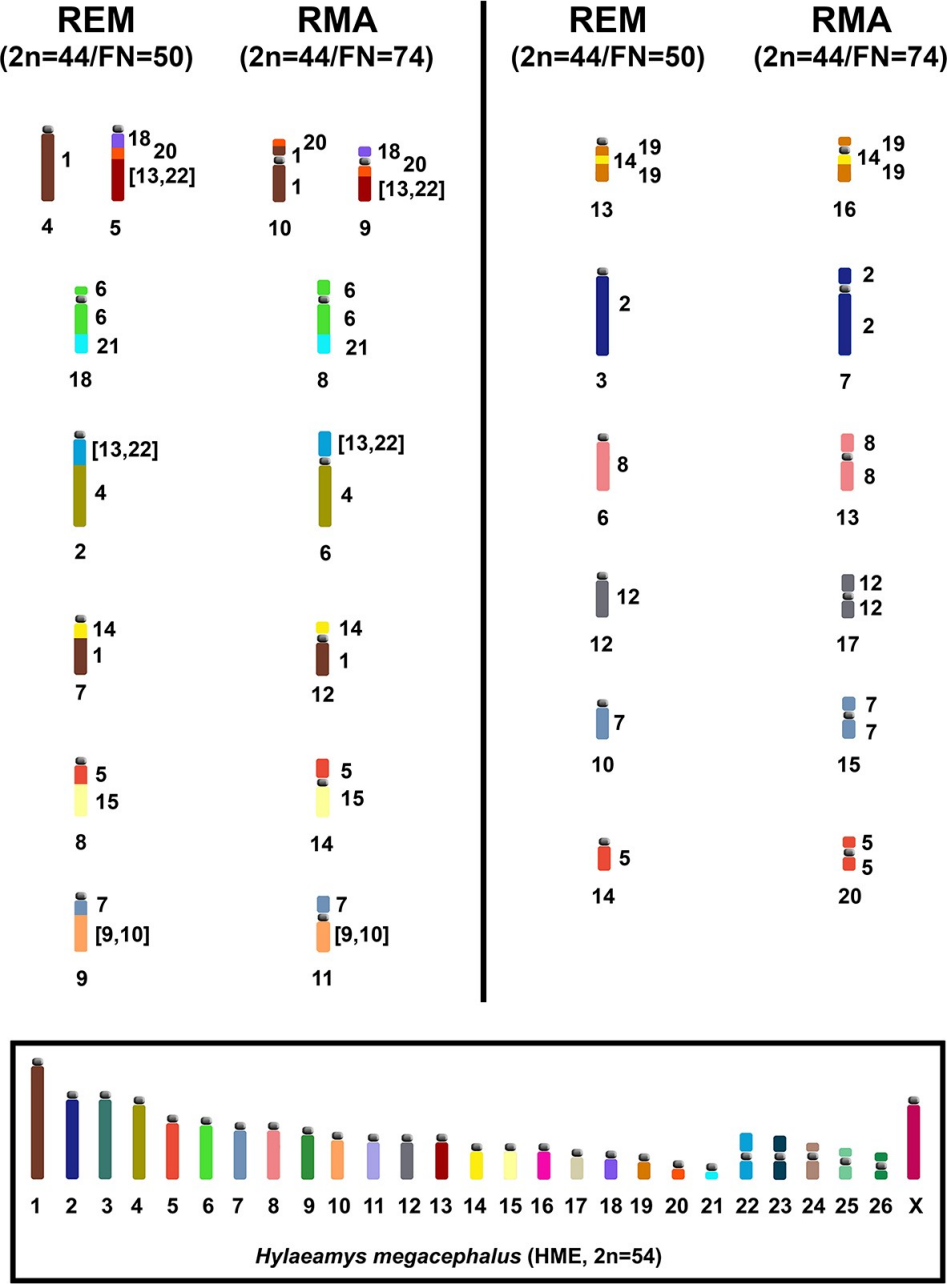
Idiograms of the haploid content of *R*. *emiliae* (REM; 2n = 44/FN = 50) and *R*. *mastacalis* (RMA; 2n = 44/FN = 74), showing chromosomes involved in their karyotypic divergence, as assessed using HME probes [31]. HME probes are shown beside the idiograms, while each chromosomal pair is identified below. Idiograms within the box correspond to the *H*. *megacephalus* karyotype elaborated by Oliveira da Silva et al. [40].

Our comparative chromosome painting analysis between *R*. *emiliae* (2n = 44/FN = 50; Fig 2A) and *R*. *mastacalis* (2n = 44/FN = 74; Fig 3A) karyotypes showed that there was no detectable difference between the species for eight autosomal pairs and the X chromosome, so these chromosomes are conserved in both species (Table 4). The divergence in FN was due to 12 pericentric inversions or centromeric repositioning and one translocation with inversion (Table 4 and Fig 6). G-banding based comparative analysis of the 12 pairs with changes in morphology (Table 4 and Figs 6 and S3) showed that there were pericentric inversions in four pairs and centromeric repositioning in eight pairs, as the latter pairs maintained the same G-banding pattern, despite their changes in chromosome morphology.

**Table 4:** Chromosomal rearrangements that differentiate the *R*. *emiliae* and *R*. *mastacalis* cytotypes, as identified by HME whole chromosome probes.

Chromosomal rearrangement	REM (2n = 44/NF = 50)	RMA (2n = 44/NF = 74)
Centromere Repositioning	2 (*HME [13,22]/4)	6 (HME 13,22/*/4)
Pericentric inversion	3 (*HME 2)	7 (HME 2*2)
Centromere Repositioning	6 (*HME 8)	13 (HME 8*8)
Centromere Repositioning	7 (*HME 14/1)	12 (HME 14/*/1)
Centromere Repositioning	8 (*HME 5/15)	14 (HME 5/*/15)
Centromere Repositioning	9 (*HME 7/[9,10])	11 (HME 7* [9,10])
Centromere Repositioning	10 (*HME 7)	15 (HME 7*7)
Centromere Repositioning	12 (*HME 12)	17 (HME 12*12)
Pericentric inversion	13 (*HME 19/14/19)	16 (HME 19/*/14/19)
Pericentric inversion	14 (*HME 5)	20 (HME 5*5)
Centromere Repositioning ^1^	18 (HME 6*6/21)	8 (HME 6*6/21)
Translocation and Pericentric inversion ^2^	5 (*HME 18/20/[13,22])	9 (HME 18/*/20a/[13,22])
	4 (*HME 1)	10 (HME 20b/1*/1)
Conserved	1 (*HME 13,22/26/25/3)	1 (*HME 13,22/26/25/3)
Conserved	11(*HME 11/[16,17])	2 (*HME 11/[16,17])
Conserved	15 (*HME 9,10)	3 (*HME 9,10)
Conserved	16 (*HME 5/11)	5 (*HME 5/11)
Conserved	17 (*HME [16,17])	4 (*HME [16,17])
Conserved	19 (HME 24*24)	18 (HME 24*24)
Conserved	20 (HME 23*23)	19 (HME 23*23)
Conserved	21(HME 18*18)	21 (HME 18*18)
Conserved	X (*HME X)	X (*HME X)

CR = Centromere Repositioning.

(*) Centromere.

^**1**^ The short arm size differs between species.

^**2**^ The HME 20 probe sequence is involved in a translocation: it hybridizes to one chromosome in *R*. *emiliae* (REM 5q interstitial) and two chromosomes in *R*. *mastacalis* (RMA 9q proximal and 10p distal).

In the other genera of Sigmodontinae rodents investigated by whole-chromosome probes, such as *Oligoryzomys* [38], *Neacomys* [39, 40], *Oecomys* [38, 42] and *Akodon* [31, 32, 34, 35], the diversity in 2n and FN was due to pericentric inversions, multiple fusion/fissions, and translocation events. However, *Rhipidomys* exhibits an unusual karyotypic evolutionary pattern. Data based on classical cytogenetics suggested that pericentric inversions are the predominant rearrangements responsible for the divergence between *R*. *emiliae* and *R*. *mastacalis* [11, 12, 16, 19, 20]. Thus, the new detection of a translocation in the present study demonstrates the great efficiency of the chromosome painting in the comparative analysis of karyotypes. It is possible that other rearrangements that are not visualized with classical methods may exist to differentiate the karyotypes of *Rhipidomys* species.

Pericentric inversions play an important role in the reorganization of rodent genomes, since they can act in reproductive isolation [48]. Comparative studies of chromosomes in primates, other mammals, and birds have shown that centromeres can change their position throughout evolution without any change in the order of DNA markers around the new centromeric location (centromeric repositioning) [49]. The most parsimonious way for this to occur would be through the inactivation of the original centromere and formation of a new centromere in another location [49, 50]. Several evolutionary studies have indicated that centromeric repositioning is not rare in karyotype evolution, and that it should be considered on equal terms with traditional chromosomal rearrangements when examining the evolution of chromosomal structure [50, 51]. As seen for inversions, the repositioning of the centromere on a chromosome provides an effective mechanism for reproductive isolation and, therefore, speciation [49]. Inversions can create linkage groups that cause sterility between hybridizing taxa, and natural selection will have a greater opportunity to decrease the frequency of interspecies mattings [52]. They also can reduce gene flow by suppressing recombination and extending the effects of linked isolation genes [53].

In the present study, we observed four pericentric inversions and eight examples of centromeric repositioning (S3 Fig). We speculate that the constancy of this type of rearrangement may be related to the process of reproductive isolation between these species.

The chromosomal evolution process that occurs in *Rhipidomys* can be classified as "karyotype orthoselection", wherein certain strains acquire a series of rearrangements of a particular type [54, 55]. There is evidence that orthoselection may be associated with specific adaptive values that have certain evolutionary connotations [56]. Some rearrangements would be selectively advantageous in mammals due to their effect on gene recombination, where the elevation of FN would increase the amount of recombination [55]. The 13 events of change in the centromeric position between REM and RMA, six of which occurred within conserved blocks and seven between syntenic blocks (Fig 6), can be understood as karyotypic orthoselection.

The phylogenetic analysis of the genus *Rhipidomys* carried out using cytochrome b showed that *R*. *macconnelli* may be one of the first species within the genus to diverge [7]. The fact that this species has 2n = 44/FN = 50 suggests that the ancestral karyotype of the genus would have 2n = 44 and a low FN, from which the high FN would be derived. This evidence is reinforced by the sharing of 2n = 44 and a predominantly acrocentric karyotype formula among most genera of the Thomasomyini tribe (*Rhipidomys*, *Thomasomys* and *Aepeomys*) [57–59]. The clade formed by *R*. *mastacalis* + *R*. *emiliae* + *R*. *ipukensis* [7] has the highest chromosomal diversity (see S1 Table). Since *R*. *emiliae* and *R*. *ipukensis* are more closely related to each other than either is to *R*. *mastacalis* and that *R*. *ipukensis* and *R*. *mastacalis* have high FN while *R*. *emiliae* has low FN, we can assume that high FN appeared independently in *R*. *ipukensis* and *R*. *mastacalis* or that the common ancestor of the three species had high FN and later it was reduced in *R*. *emiliae*.

### Characters shared among species of Sigmodontinae subfamily

Representatives of two tribes of the Sigmodontinae subfamily (Oryzomyini and Akodontini) have been studied with probes from *Hylaeamys megacephalus* (HME) [30–32, 40–42]. In the present study, we extended this analysis to two species of the Thomasomyini tribe. We found that these species have some conserved chromosomes or chromosomal syntenies that are considered ancestral to the subfamily Sigmodontinae, namely HME 8, 6/21, 7/[9,10], 11/[16,17], 19/14/19, 20/[13,47], and 24. The chromosomes 15 and 26 are found as single chromosomes in Oryzomyini and Akodontini, but are fused in Thomasomyini, while the associations 1/12 and 5/[16,17] are found in the other two tribes, but split in Thomasomyini (Tables 5 and S2) [32, 39, 40]. To these, we now add the HME 1a/1b, which is shared by the Oryzomyini, Akodontini, and Thomasomyini tribes.

**Table 5:** Syntenic blocks conserved in the genomes of species that have been hybridized with *H*. *megacephalus* probes [32, 39, 40].

HME	8	15	24	26	1a, 1b	1/12	6/21	7/ (9,10)	5/16,17	11/ (16,17)	19/14/19	20/(13,22)
**CLA**	-	+	+	+	+	+	+*	+*	+	+	+*	+
**NSP-A**	-	+	+	-	+	-	+*	+*	-	-	+*	+
**NSP-B**	-	+	+	+	+	+	+*	+*	-	-	-	+
**NSP-C**	-	+	+	-	+	-	+*	+*	-	-	+*	+
**NSP-D**	-	+	+	-	+	-	+*	+*	-	-	+*	+
**NSP-E**	-	+	+	+	+	-	+*	+*	-	-	-	+
**NPA**	-	+	+	+	+	-	+*	+*	-	+	+	+
**NAM**	-	+	+	+	+	-	+*	+*	-	-	-	+
**OCA**	+	+	+	-	-	-	-	-	-	-	-	+
**OPA**	+	+	+	+	-	-	-	-	-	-	+*	+*
**TNI**	+	+	+	+	+	-	+	-	-	+	+*	+
**NLA**	+	-	-	+	+	+	+	+	-	+	+	+
**AMO**	+	-	-	+	+	+	+	+	+	+	+	+
**ASP**	+	-	-	+	+	+	+	-	+	+	+	+
**OAM**	+	+	+	+	+	-	+	+	-	+	+	+
**BBR**	**+**	-	-	-	+	-	+	+	-	-	+*	**+**
**REM**	+	-	+	-	+	-	+	+	-	+	+	+
**RMA**	+	-	+	-	+	-	+	+	-	+	+	+

(+) Presence; (-) absence of character;

(*) Present in a reorganized form;

**Tribe Oryzomyini**: CLA = *Cerradomys langguthi* [30]; OCA = *Oecomys catherinae* [38]; NSP-A = *Neacomys* sp. A, NSP-B = *Neacomys* sp. B [39]; NSP-C = *Neacomys* sp. C, NSP-D = *Neacomys* sp. D, NPA = *N*. *paracou*, NSP-E = *Neacomys* sp. E, NAM = *N*. *amoenus* [40]; OPA *= O*. *paricola* [42]. **Tribe Akodontini:** TNI *= Thaptomys nigrita*, AMO *= Akodon montensis* [31]; ASP = *Akodon* sp., NLA = *Necromys lasiurus* [32]; OAM = *Oxymycterus amazonicus*, BBR = *Blarinomys breviceps* [41]. **Tribe Thomasomyini**: REM = *Rhipidomys emiliae*, RMA = *R*. *mastacalis* (present study).

**Fig 7:**
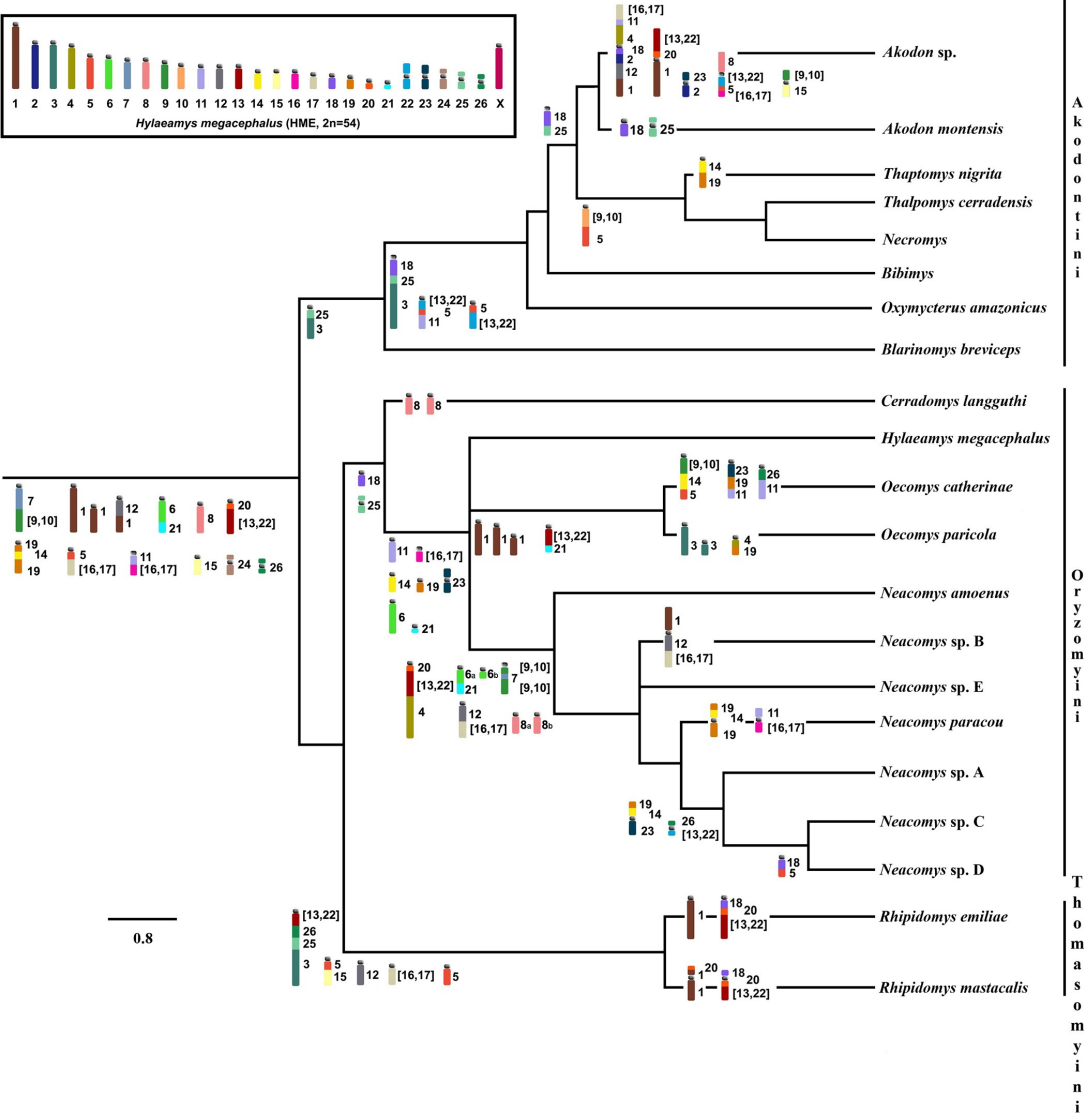
Chromosomal evolution in the tribes Oryzomyini, Akodontini, and Thomasomyini. The phylogeny was based on [40] and [41]. The chromosomes colors and morphology refer to the HME karyotype inside the box is the idiogram of the HME karyotype [41], from which the whole chromosome probes were made.

The Fig 7 shows the syntenic blocks mentioned in the genomes of species at Table 5, in a phylogenetic perspective.

The HME 8, 6/21, 7/[9,10], 11/(16,17), 19/14/19, and 24 (acrocentric) syntenies remain as independent blocks in the *R*. *emiliae* and *R*. *mastacalis* karyotypes, and are not associated with other probes (Figs 2A and 3A). The HME 20/(13,22) association is fused to one of the HME 18 segments, originating the HME 18/20/(13,22) association. In *R*. *emiliae*, the HME 20 probe is found as a single block, as has been seen in most species analyzed with the same probe (S2 Table). In *R*. *mastacalis*, by contrast, it is fragmented into two blocks of different sizes and the smallest segment resulting from this fission is associated with one of the two HME 1 signals, originating the HME 20/1/*/1 (RMA 10) association. Therefore, it is possible that, in *Rhipidomys*, a single signal for HME 20 (REM 5 interstitial) is the original chromosomal condition (Fig 2A), while a dual signal is the derivative form, arising from translocation, and is probably an autapomorphic characteristic of *R*. *mastacalis* (Fig 3A).

The HME 26, 15, and the syntenic blocks 5/(16,17), 1/12, have been described as ancestral characters, but are absent in *Rhipidomys* in this form of organization (Table 5). The HME 26 segment underwent tandem fusion, resulting in the HME association (13,22)/26/25/3 (REM 1 and RMA 1). The association HME 5/(16,17) is shared only among *Cerradomys langguthi* (CLA) + *Akodon montensis* (AMO) + *Akodon* sp. (ASP); in *Rhipidomys* it suffered a fission that generated two independent units HME (16,17) (REM 17 and RMA 4) and HME 5. It is therefore difficult to define what region would be homologous to this region of HME 5 in *Rhipidomys*, since this pair is the most fragmented in *Rhipidomys* and other species.

The HME 15 (not associated) has been described as a symplesiomorphic character (Table 5). This configuration is present in Oryzomyini and, among the Akodontini, it is observed only in *Thaptomys nigrita* (TNI) and *Oxymycterus amazonicus* (OAM). In the other Akodontini and Thomasomyini, it is associated with another HME probe. In *R*. *emiliae* and *R*. *mastacalis*, this block is associated with one of the HME 5 segments, giving rise to the association HME 5/15 (REM 8 and RMA 14).

The HME 1/12 association suffered a fission to produce two segments, one corresponding to HME 12 (REM 12 and RMA 17) and the other to HME 1. The latter associated with one of the HME 14 fragments, giving rise to the HME 14/1 (REM 7 and RMA 12). Pereira et al. [32] compared their results with the hybridization data obtained using *Mus musculus* (MMU) probes [33], and found that the association HME 1/12 (MMU 3/18) is absent in *Oligoryzomys*. Later studies found that this association is also absent in most species of tribe Oryzomyini, occurring only in *Neacomys* sp. B (NSP-B) and *C*. *langguthi*. This association is also absent in *Rhipidomys*, suggesting it is an exclusive character of the Akodontini tribe. Its presence in *Neacomys* sp. B and *C*. *langguthi* would then be a homoplasy. We observed that in all of these analyzed species, the HME 1 probe shows at least two signals (three in the *Oecomys* species already analyzed to date), which reinforces the proposal that in *H*. *megacephalus* this chromosome is the result of fusion [31].

The association HME 25/3 that has been suggested as an exclusive ancestral character of Akodontini is also present in *Rhipidomys*. Fissioned HME 18 (18a and 18b, S2 Table) is a character that is also shared among these tribes. Thus, we speculate that these characters were present in the ancestral karyotype of Sigmodontinae and appear in a derived condition in Oryzomyini. Alternatively, it could be a synapomorphy (or even a homoplasy) only between Akodontini and Thomasomyini. Two other associations of *Rhipidomys* are shared with some Akodontini species: HME 5/11 (REM 16, RAM 5, NLA 1 [*Necromys Lasiurus*], ASP 3 [*Akodon* sp.], OAM 2 [*O*. *amazonicus*]) and 20/1 (RMA 10, ASP 1 [*Akodon* sp], AMO 4 [*A*. *nontensis*], TNI 4 [*T*. *nigrita*], Table 5).

The associations HME (13,22)/26/25/3, 18/20/(13,22), (13,22)/4, 14/1, and 5/15 were found exclusively in *Rhipidomys*, and can be considered synapomorphies for the genus. The future inclusion of other representatives of the Thomasomyini tribe in comparative analyses with HME probes may shed further light on whether these characters are exclusive to *Rhipidomys*, or if they can be considered as signatures for the tribe.

## Conclusions

The results of this work demonstrate that the rearrangements responsible for genomic diversification between the karyotypes of *R*. *emiliae* and *R*. *mastacalis* and, possibly, of the other species of *Rhipidomys*, involve a combination of translocations, centromeric repositioning and pericentric inversions. The translocation found herein could not be easily detected through classical cytogenetics, and thus our work demonstrates the usefulness of chromosomal painting for such analyses. Comparative analysis with other species of Sigmodontinae shows that *Rhipidomys* (Thomasomyini) shares the synthetic blocks HME 8, 6/21, 20/[13,22], 5/11, 7/[9,10] and 19/14/19 and 24 with species from the Akodontini and Oryzomyini tribes. We also suggest that HME 25/3 association and HME 18a and 18b may be a synapomorphies between Akodontini and Thomasomyini; and fissioned HME 1 may be a symplesiomorphic character for the Sigmodontinae subfamily. It will be interesting to expand the use of HME probes to other species of *Rhipidomys*, in order to define chromosomal signatures that may be used to elucidate the taxonomic and phylogenetic relationships between species of this genus and enable a better reconstruction of the ancestral karyotype for the Sigmodontinae subfamily.

## Supporting information

S1 Fig A simplified phylogeny of Sigmodontinae modified from Gonçalves et al. (2020)* to show the relationships among the tribes. In bold letters, the tribes Oryzomyini (*Hylaeamys*) and Thomasomyini (*Rhipidomys*). *Gonçalves, P.R.; Christoff, A.U.; Machado, L.F.; Bonvicino, C.R.; Peters, F.B.; Percequillo, A.R. Unraveling deep branches of the Sigmodontinae tree (Rodentia: Cricetidae) in Eastern South America. Journal of Mammalian Evolution **2020**, 27:139–160.
(TIF)

S2 Fig Hybridization of all whole chromosome probes of HME in *Rhipidomys* species. A) *R*. *emiliae* (2n = 44/ FN = 50). B) *R*. *mastacalis* B (2n = 44 / FN = 74). Each probe refers to a chromosome pair, with the exception of HME [9,10], [16,17], [13,22] which are equivalent to 2 pairs of chromosomes each. Avidin-Cy3 (red) and avidin-FITC (green).
(TIF)

S3 Fig Comparison of G-banded haploid sets of *Rhipidomys emiliae* (REM; 2n = 44/FN = 50) and *R*. *mastacalis* (RMA; 2n = 44/FN = 74), showing chromosomes involved in the karyotypic divergence, based on HME probes [30]. HME probes are shown beside the chromosomes, while the identification of the chromosomal pair is shown below.
(TIF)

S1 Table Compilation of *Rhipidomys* cytogenetic data. **Abbreviations:** Brazilian states: Bahia (BA), Ceará (CE), Goiás (GO), Espírito Santo (ES), Mato Grosso (MT), Minas Gerais (MG), Pará (PA), Pernambuco (PE), and Piauí (PI); National Forest (FLONA). Natural Heritage Private Reserve (RPPN); Biological Reserve (ReBio); National Park (PARNA). Ecological Station (ESEC); State Park (PE); diploid number (2n); and autosomal fundamental number (FN).
(DOCX)

S2 Table FISH signals detected for Sigmodontinae species based on hybridization with *Hylaeamys megacephalus* (HME) whole-chromosome probes [30]. (DOCX)
