# Supplementary figures and images for: Comparative genomic mapping reveals mechanisms of chromosome diversification in Rhipidomys species (Rodentia, Thomasomyini) and syntenic relationship between species of Sigmodontinae
Source: PLoS One. 2021 Oct 11;16(10):e0258474. doi: 10.1371/journal.pone.0258474 (PMC8504764; doi:10.1371/journal.pone.0258474)

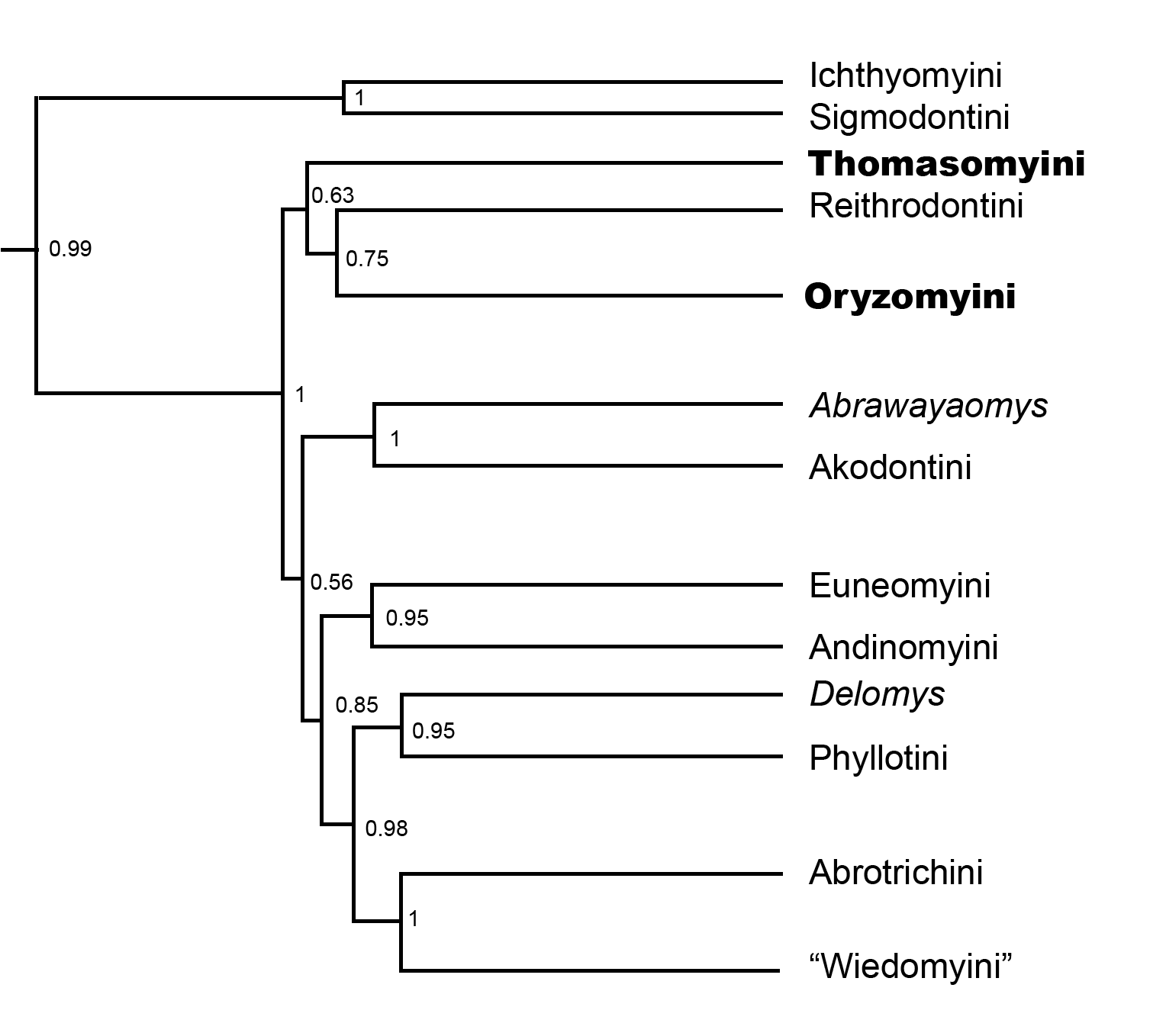

Supplement: S1 Fig — In bold letters, the tribes Oryzomyini (Hylaeamys) and Thomasomyini (Rhipidomys). *Gonçalves, P.R.; Christoff, A.U.; Machado, L.F.; Bonvicino, C.R.; Peters, F.B.; Percequillo, A.R. Unraveling deep branches of the Sigmodontinae tree (Rodentia: Cricetidae) in Eastern South America. Journal of Mammalian Evolution 2020, 27:139–160. (TIF) [file pone.0258474.s001.tif]

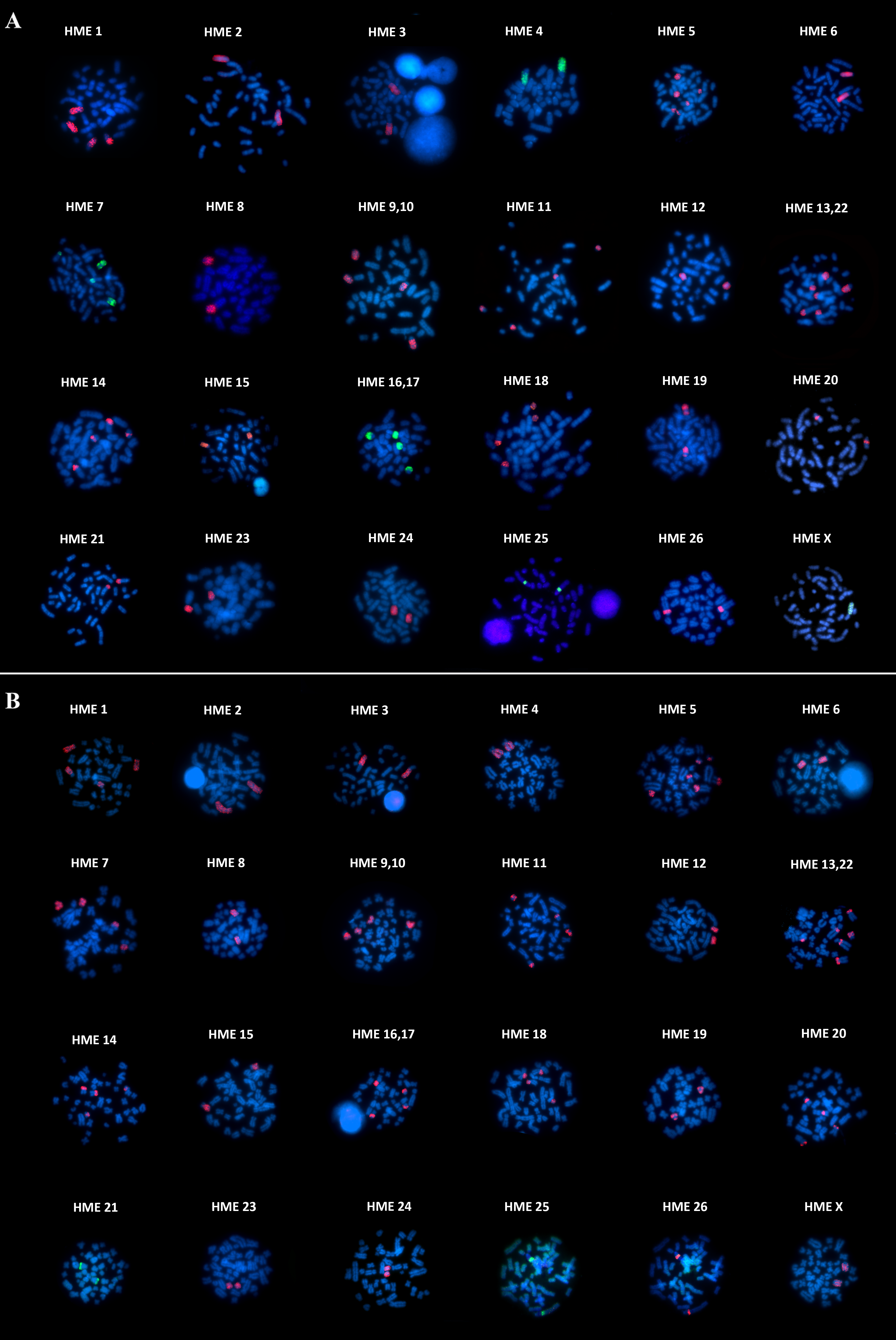

Supplement: S2 Fig — Each probe refers to a chromosome pair, with the exception of HME [9,10], [16,17], [13,22] which are equivalent to 2 pairs of chromosomes each. Avidin-Cy3 (red) and avidin-FITC (green). (TIF) [file pone.0258474.s002.tif]

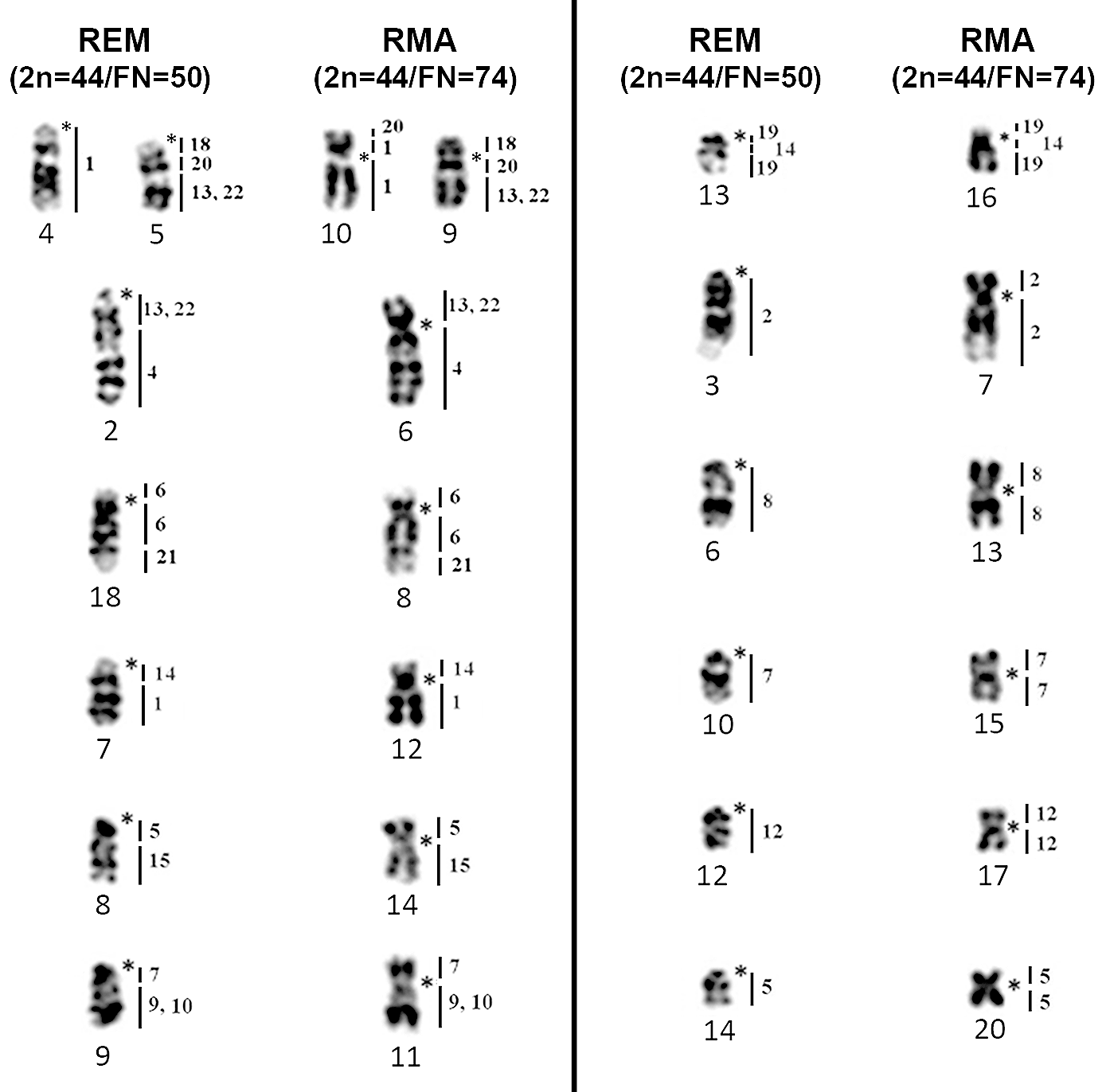

Supplement: S3 Fig — HME probes are shown beside the chromosomes, while the identification of the chromosomal pair is shown below. (TIF) [file pone.0258474.s003.tif]
