# Supplementary material for: Comparative genomic mapping reveals mechanisms of chromosome diversification in Rhipidomys species (Rodentia, Thomasomyini) and syntenic relationship between species of Sigmodontinae
Source: PLoS One. 2021 Oct 11;16(10):e0258474. doi: 10.1371/journal.pone.0258474 (PMC8504764; doi:10.1371/journal.pone.0258474)
Supplement: S1 Table — Abbreviations: Brazilian states: Bahia (BA), Ceará (CE), Goiás (GO), Espírito Santo (ES), Mato Grosso (MT), Minas Gerais (MG), Pará (PA), Pernambuco (PE), and Piauí (PI); National Forest (FLONA). Natural Heritage Private Reserve (RPPN); Biological Reserve (ReBio); National Park (PARNA). Ecological Station (ESEC); State Park (PE); diploid number (2n); and autosomal fundamental number (FN). (DOCX) [file pone.0258474.s004.docx]

**S1 Table. Compilation of *Rhipidomys* cytogenetic data.** Brazilian states: Bahia (BA), Ceará (CE), Goiás (GO), Espírito Santo (ES), Mato Grosso (MT), Minas Gerais (MG), Pará (PA), Pernambuco (PE), Piauí (PI). National Forest (FLONA). Natural Heritage Private Reserve (RPPN). Biological Reserve (ReBio). National Park (PARNA). Ecological station (ESEC). State park (PE). Diploid number (2n), Autosomal fundamental number (FNa), G-banding (G), C-banding (C), R-banding (R), NOR staining (NOR), Telomeric FISH (Tel-FISH).

| **Species** | **2n** | **FN** | **Cytogenetic** | **Locality** | **References** |
| --- | --- | --- | --- | --- | --- |
| *R. couesi* **^a^** | 44 | 48 | Conventional staining | Venezuela: Anzoátegui, Cueva del Agua | [1] |
| *R. latimanus* | 44 | 48 | Conventional staining | Colômbia: Peñas Blancas | [2] |
| *R. itoan* | 44 | 48-50 | Conventional staining, NOR | Brazil: RJ | [3] |
| *R. itoan* **^b^** | 44 | 49 | G, C, R, NOR | Brazil: SP, Casa Grande | [4] |
| *R. itoan* | 44 | 50 | Conventional staining | Brazil: SP, Cotia | [5] |
| *R. itoan* | 44 | 50 | Conventional staining | Brazil: SP, PE Serra do Mar, Núcleo Santa Virgínia | [6] |
| *R. leucodactylus* | 44 | 46 | Conventional staining | Brazil: AM, Ipixuna, Condor, | [7] |
| *R. leucodactylus* | 44 | 48 | Conventional staining | Brazil: RO, Rio Jamari, Usina Hidrelétrica de Samuel | [8] |
| *R. leucodactylus* | 44 | 48 | C, NOR, Tel-FISH | Brazil: GO, Ipameri; Caldas Novas; Corumbaíba | [9] |
| *R. leucodactylus* **^c^** | 44 | 52 | G, C, NOR, Tel-FISH | Brazil: MT, Aripuanã | [10] |
| *R. macrurus* **^d^** | 44 | 48 | G, C, R, NOR | Brazil: GO, DF, Granja do Ipê | [4] |
| *R. macrurus* | 44 | 48 | Karyotype unavailable | Brazil: TO, Ipueiras | [11] |
| *R. macrurus* | 44 | 48 | Conventional staining | Brazil: BA, Lençóis, Remanso (Chapada Diamantina) | [12] |
| *R. macrurus* **^d^** | 44 | 49 | G, C, R, NOR | Brazil: GO, DF, ReBio de Águas Emendadas | [4] |
| *R. macrurus* | 44 | 50 | G | Brazil: PI, Bom Jesus, ESEC Uruçuí-Una; | [13] |
| *R. macrurus* | 44 | 50 | Karyotype unavailable | Brazil: MG, Nova Ponte, Mata do Vasco | [14] |
| *R. macrurus* | 44 | 50 | Conventional staining | Brazil: MG, Nova Ponte | [5] |
| *R. macrurus* | 44 | 50 | Conventional staining | Brazil: MS, Dourados | [5] |
| *R. macrurus* | 44 | 50 | G | Brazil: PI; Caracol, PARNA Serra das Confusões | [13] |
| *R. macrurus* **^e^** | 44 | 52 | C, NOR, Tel-FISH | Brazil: GO, Serra da Mesa, 20 km NW Colinas do Sul | [9] |
| *R. macconnelli* | 44 | 50 | Conventional staining | Venezuela, Bolivar, La Escalera | [1] |
| *R. gardneri* | 44 | 50 | Conventional staining | Brazil: Acre, Porto Walter | [7] |
| *R. tribei* **^f^** | 44 | 50 | Karyotype unavailable | Brazil: ES, Ibitirama, PARNA Caparaó | [14] |
| *R. tribei* | 44 | 50 | Conventional staining, NOR | Brazil, ES, Ibitirama | [5] |
| *R. tribei* **^g^** | 44 | 50 | Conventional staining | Brazil: ES, Muqui, Fazenda Recanto | [14] |
| *R. tribei* | 44 | 50 | Conventional staining, NOR | Brazil, ES, Muqui | [5] |
| *R. tribei* **^b^** | 44 | 50 | G, C | Brazil: ES, Hotel Fazenda Monte Verde, 24 km SE de Venda Nova | [8] |
| *R. cariri* **^h^** | 44 | 50 | Karyotype unavailable | Brazil: PE, Buíque, PARNA Catimbau | [14] |
| *R. cariri* | 44 | 50 | Conventional staining | Brazil: PE, Buíque | [5] |
| *R. cariri cariri* | 44 | 50 | Karyotype unavailable | Brazil: CE, Crato, Chapada do Araripe | [14] |
| *R. cariri* | 44 | 50 | Conventional staining, NOR | Brazil, CE, Crato | [5] |
| *R. cariri* **^b^** | 44 | 50 | Conventional staining | Brazil: PE, PARNA do Catimbau, Buíque | [15] |
| *R. emiliae* | 44 | 50 | G, C, Tel-FISH, Zoo-FISH | Brazil: Pa, Parauapebas, FLONA de Carajás | **Present study** |
| *R. emiliae* | 44 | 50 | G, C, Tel-FISH, Zoo-FISH | Brazil: Pa, Marabá, FLONA de Tapirapé-Aquiri | **Present study** |
| *R. emiliae* **^i^** | 44 | 50 | Karyotype unavailable | Brazil: MT, Ribeirão Cascalheiras, Fazenda Noruimbá | [14] |
| *R. emiliae* **^j^** | 44 | 52 | C, NOR, Tel-FISH | Brazil: PA, Melgaço, FLONA de Caxiuanã | [9] |
| *R. emiliae* | 44 | 52 | Conventional staining | Brazil: MT, Ribeirão Cascalheira | [5] |
| *R. emiliae* | 44 | 52 | Conventional staining | Brazil: MT, Barra do Garças | [5] |
| *R. emiliae* **^k^** | 44 | 52 | G, C, NOR, Tel-FISH | Brazil: MT, Vila Rica | [10] |
| *Rhipidomys* sp. | 44 | 48 | G, C, NOR, Tel-FISH | Brazil: MG, Berilo | [16] |
| Unidentified specimen | 44 | 48 | Karyotype unavailable | Brazil: MG, Formoso, PARNA Grande Sertão Veredas | [14] |
| Unidentified specimen | 44 | 48 | Karyotype unavailable | Brazil: TO, Ponte Alta do Tocantins, ESEC Serra Geral do Tocantins | [14] |
| *Rhipidomys* sp.1 | 44 | 50 | Conventional staining, C | Brazil: MG, Coronel Murta | [5] |
| *Rhipidomys* sp.3 | 44 | 50 | Karyotype unavailable | Brazil: MG, Coronel Murta, Ponte do Colatino | [14] |
| *Rhipidomys* sp.1 | 44 | 50 | Conventional staining, | Brazil: BA, Andaraí | [5] |
| *Rhipidomys* sp.3 | 44 | 50 | Conventional staining | Brazil: BA, Andaraí, Fazenda Santa Rita | [14] |
| Unidentified specimen | 44 | 50 | Conventional staining | Brazil: SP, Cotia, Reserva do Morro Grande | [14] |
| *Rhipidomys* sp. A (Hybrid) | 44 | 61 | G, C, NOR, Tel-FISH | Brazil: BA, Morro do Chapéu | [10] |
| Unidentified specimen | 44 | 50 | Karyotype unavailable | Brazil: TO, Jalapão, Lagoa do Toca | [14] |
| *R. mastacalis* **^l^** | 44 | high | Karyotype unavailable | Brazil, PE, Serra dos Cavalos, 13 km ESE São Caitano | [8] |
| *R. mastacalis* **^m^** | 44 | 72 | Karyotype unavailable | Brazil: CE, Ipú, Serra de Ipiapaba | [13] |
| *R. mastacalis* **^m^** | 44 | 72 | Karyotype unavailable | Brazil: PE, Brejo da Madre de Deus, RPPN Fazenda Bituri | [13] |
| *R. mastacalis* **^m^** | 44 | 72 | G | Brazil: BA, Una, ReBio de Una | [13] |
| *R. mastacalis* **^m^** | 44 | 70 | G | Brazil: BA, Ilhéus, Centro Experimental Almada | [13] |
| *R. mastacalis* **^m^** | 44 | 70 | G | Brazil: BA, Jussari, RPPN Serra do Teimoso | [13] |
| Unidentified specimen | 44 | 72 | Conventional staining, C | Brazil: BA, Una | [14] |
| *R.* aff. *mastacalis* 1 | 44 | 72 | Conventional staining | Brazil: BA, Una | [5] |
| *R.* aff. *mastacalis* 1 | 44 | 72 | Conventional staining | Brazil: BA, Itajú do Colônia | [5] |
| *R. mastacalis* | 44 | 74 | Karyotype unavailable | Brazil: ES, Águia Branca | [14] |
| *R. mastacalis* | 44 | 74 | Conventional staining | Brazil: ES, Águia Branca | [5] |
| *R. mastacalis* | 44 | 74 | Conventional staining, C | Brazil: ES, Cariacica, ReBio Duas Bocas | [14] |
| *R.* aff. *mastacalis 2* | 44 | 74 | Conventional staining, C, NOR | Brazil: ES, Cariacica | [5] |
| *R. mastacalis* | 74 | 74 | Karyotype unavailable | Brazil: ES, Muqui, Fazenda Recanto | [14] |
| *R.* aff. *mastacalis 2* | 74 | 74 | Conventional staining, C, NOR | Brazil: ES, Muqui | [5] |
| Unidentified specimen | 44 | 74 | Karyotype unavailable | Brazil: ES, Jacutinga, Região do Alto Misterioso | [14] |
| *R.* aff. *mastacalis 2* | 44 | 74 | Conventional staining, C, NOR | Brazil: ES, Santa Tereza | [5] |
| *R. mastacalis* | 44 | 74 | Karyotype unavailable | Brazil: MG, Lagoa Santa | [8] |
| *R. mastacalis* | 44 | 74 | G, C | Brazil: BA, Una, Fazenda Unacau, 8km São José | [8] |
| *R. mastacalis* | 44 | 74 | Karyotype unavailable | Brazil: ES, Cariacica, ReBio Duas Bocas | [17] |
| *R. mastacalis* | 44 | 74 | G, C, Tel-FISH, Zoo-FISH | Brazil: MG, Fazenda Palmares, Padre Paraíso | **Present study**, [18] |
| *R. mastacalis* | 44 | 74 | G, C, Tel-FISH, Zoo-FISH | Brazil: MG, Diamantina, Fazenda Santa Cruz | **Present study**, [18] |
| *R. mastacalis* cytotype 2 | 44 | 76 | C, NOR, Tel-FISH | Brazil: GO, Serra da Mesa, 20 km NW Colinas do Sul;  40 km SW Minaçú; 40 km NE Uruaçú; 55 km N Niquelândia | [9] |
| *R. mastacalis* cytotype 1 | 44 | 80 | C, NOR, Tel-FISH | Brazil: GO, Serra da Mesa, 20 km NW Colinas do Sul;  40 km NE Uruaçú | [9] |
| *R. ipukensis* | 44 | 80 | Conventional staining | Brazil: TO, Peixe | [5] |
| *R. nitela* | 48 | 67 | G, C, R | Guiana Francesa, La Trinité | [19] |
| *R. nitela* | 48 | 68 | C, NOR, Tel-FISH | Brazil: RR, Surumú | [9] |
| *R. nitela* **^n^** | 50 | 71 | G, C, NOR, Tel-FISH | Brazil: AM, Manaus | [10] |
| *R. nitela* **^n^** | 50 | 72 | G, C, NOR, Tel-FISH | Brazil: AM, Manaus | [10] |
| *R. nitela* | 48 | 66 | Conventional staining | Venezuela: Bolívar, San Ignácio | [14] |

**^a^** Identified in the original article as *R. sclateri* and reviewed by Tribe [1];

**^b^** Identified in the original article as *Rhipidomys* sp. and reviewed by Tribe [2];

**^c^** Identified in the original article as *R.* cf. *mastacalis* and reviewed by Tribe [2];

**^d^** Identified in the original article as *Rhipidomys* sp. and reviewed by Tribe [1];

**^e^** Identified in the original article as *R. leucodactylus* cytotype 1 and reviewed by Tribe [2];

**^f^** Identified in the original article as *R. macrurus* and reviewed by Tribe [2];

**^g^** The author did not identify at the species level in the original article and Tribe [2] described it as *R. tribei*;

**^h^** The author did not identify at the species level in the original article and Tribe [2] described it as *R. cariri*;

**^i^** Identified in the original study as *Rhipidomys* sp., however Costa et al. [4] and Tribe [2] assign the collecting locality of the specimen to the range of *R. emiliae.*

**^j^** Identified in the original article as *R. leucodactylus* cytotype 1 and reviewed by Tribe [2];

**^k^** Identified in the original article as *R.* cf. *mastacalis* and reviewed by Tribe [2];

**^l^** Identified in the original article as *R. cearanus*, however this name is currently considered a synonymy of *R. mastacalis* [3];

**^m^** Previously assigned with 2n = 44/FNa = 70 [16], however we corrected to 2n = 44/FNa = 72 [13];

**^n^** Identified in the original article as *Rhipidomys* sp B and reviewed by Tribe [2].

**References**

1. Aguilera, M.; Pérez-Zapata, A.; Martino, A.; Barros, M.A.; Patton, J. Karyosystematics of *Aepeomys* and *Rhipidomys* (Rodentia, Cricetidae). *Acta Cient. Venez*. **1994**, 45: 247–248.
2. Gardner, A.L.; Patton, J.L. Karyotypic variation in Oryzomyini rodents (Cricetinae) with comments on chromosomal evolution in the Neotropical cricetinae complex. *Occas. Papers Mus. Zool. Louisiana State Univ*. **1976**, 49: 1- 48.
3. Costa, B.M.A.; Geise, L.; Pereira, L.G.; Costa, L.P. Phylogeography of *Rhipidomys* (Rodentia: Cricetidae: Sigmodontinae) and the description of two new species from southeastern Brazil. *J. Mammal*. **2011**, 92: 945–962. <https://doi.org/10.1644/10-MAMM-A-249.1>
4. Svartman, M.; Almeida, E.J.C. Pericentric inversion and X chromosome polymorphism in *Rhipidomys* sp. (Cricetidae, Rodentia) from Brazil. *Caryologia* **1993**, 46: 219-225. <https://doi.org/10.1080/00087114.1993.10797262>
5. de Carvalho, A.H. Evolução Molecular e Cariotípica em *Rhipidomys* Tschudi, 1845 (Rodentia, Cricetidae). PhD dissertation, Universidade Federal do Espírito Santo, Brasil, 2017.
6. Di-Nizo, C.; Neves, C.; Fernando Vilela, J.; Silva, M. New karyologycal data and cytotaxonomic considerations on small mammals from Santa Virgínia (Parque Estadual da Serra do Mar, Atlantic Forest, Brazil). *Comp. Cytogenet.* **2014**, 8(1): 11-30. <https://doi.org/10.3897/compcytogen.v8i1.6430> PMID: 24744831
7. Patton, J.L.; da Silva, M.N.F.; Malcolm, J.R. Mammals of the rio Juruá and the evolutionary and ecological diversification of Amazonia. *Bull Am Mus Nat Hist*. **2000**, 244: 1-306. [https://doi.org/10.1206/0003-0090(2000)244<0001:MOTRJA>2.0.CO;2](https://doi.org/10.1206/0003-0090(2000)244%3c0001:MOTRJA%3e2.0.CO;2)
8. Zanchin, N.I.T.; Langguth, A.; Mattevi, M.S. Karyotypes of Brazilian species of *Rhipidomys* (Rodentia, Cricetidae). *J. Mamm*. **1992**, 73(1): 120-122. <https://doi.org/10.2307/1381872>
9. Andrades-Miranda, J.; Lima-Rosa, C.A.V; Sana, D.A; Nunes, A.P; Mattevi, M.S. Genetic studies in representatives of genus *Rhipidomys* (Rodentia: Sigmodontinae) from Brazil. *Acta Theriol*. **2002**, 47: 125–135. <https://doi.org/10.1007/BF03192453>
10. Silva, M.J.; Yonenaga-Yassuda, Y. Autosomal and sex chromosomal polymorphisms with multiple rearrangements and a new karyotype in the genus *Rhipidomys* (Sigmodontinae, Rodentia). *Hereditas* **1999**, 131(3): 211-220. <https://doi.org/10.1111/j.1601-5223.1999.00211.x> PMID: 10783531.
11. Saranholi, B.H.; Fonseca, R.C.B.; Lima, J.F.S. Karyologic Survey of not Flying Small Mammals from Tocantins, Brazil. *Estud. Biol*. **2008**, 30(70/71/72): 91-97. <https://doi.org/10.7213/reb.v30i70/72.22812>
12. Pereira, L.G.; Geise, L. Karyotype composition of some rodents and marsupials from Chapada Diamantina (Bahia, Brazil). *Braz. J. Biol*. **2007**, 67(3): 509-518. <https://doi.org/10.1590/S1519-69842007000300016> PMID: 18094834
13. Sousa, M.A.N. Pequenos mamíferos (Didelphimorphia, Didelphidae e Rodentia, Sigmodontinae) de algumas áreas da Caatinga, Cerrado, Mata Atlântica e Brejo de Altitude do Brasil: Considerações citogenéticas e geográficas. PhD Thesis. Universidade de São Paulo, Brasil, **2005**.
14. Thomazini, N.B. Correlação entre estrutura cariotípica e filogenia molecular em *Rhipidomys* (Cricetidae, Rodentia) do leste do Brasil. M.Sc dissertation, Universidade Federal do Espírito Santo, Brasil, 2009.
15. Geise, L.; Astúa, D.; Paresque, R.; Shirai, L.; Sebastião, H.; Marroig, G. Non-volant mammals, Parque Nacional do Catimbau,Vale do Catimbau, Buíque, state of Pernambuco, Brazil, with karyologic data. *Check List* **2010**; 6(1): 180-186. <https://doi.org/10.15560/6.1.180>
16. de Carvalho, A.H.; Lopes, M.O.G.; Svartman, M. A new karyotype for *Rhipidomys* (Rodentia, Cricetidae) from Southeastern Brazil. *Comp. cytogenet*. **2012**, 6(3): 1-11. <https://doi.org/10.3897/compcytogen.v6i3.2432>
17. Paresque, R.; Souza, W.P.; Mendes, S.L.; Fagundes, V. Composição cariotípica da fauna de roedores e marsupiais de duas áreas de Mata Atlântica do Espírito Santo, Brasil. *Bol. Mus. Biol. Mello Leitão (N. Sér.)* **2004**, 17: 5-33.
18. Geise, L.; Pereira, L.G.; Astúa, D.; Aguieiras, M.; Lessa, L.G.; Asfora, P.H.; Dourado, F.; Esberárd, C.E.L. Terrestrial mammals of the Rio Jequitinhonha River Basin, Brazil: a transition area between Atlantic Forest and Cerrado. *Mastozool Neotrop.* (Versión impresa) 2017, 24(1): 95-119.
19. Volobouev, V.T.; Catzeflis, F.M. Chromosome banding analysis (G-, R- and C bands) of *Rhipidomys* *nitela* and a review of the cytogenetics of *Rhipidomys* (Rodentia, Sigmodontinae). *Mammalia* **2000**, 64(3): 353-360. <https://doi.org/10.1515/mamm.2000.64.3.353>
